# Silencing miR-21-5p in sensory neurons reverses neuropathic allodynia via activation of TGF-**β**–related pathway in macrophages

**DOI:** 10.1172/JCI164472

**Published:** 2023-06-01

**Authors:** Lynda Zeboudj, George Sideris-Lampretsas, Rita Silva, Sabeha Al-Mudaris, Francesca Picco, Sarah Fox, David Chambers, Marzia Malcangio

**Affiliations:** Wolfson Centre for Age Related Diseases, King’s College London, London, United Kingdom.

**Keywords:** Immunology, Neuroscience, Cellular immune response, Macrophages, Pain

## Abstract

Neuropathic pain remains poorly managed by current therapies, highlighting the need to improve our knowledge of chronic pain mechanisms. In neuropathic pain models, dorsal root ganglia (DRG) nociceptive neurons transfer miR-21 packaged in extracellular vesicles to macrophages that promote a proinflammatory phenotype and contribute to allodynia. Here we show that miR-21 conditional deletion in DRG neurons was coupled with lack of upregulation of chemokine CCL2 after nerve injury and reduced accumulation of CCR2-expressing macrophages, which showed TGF-β–related pathway activation and acquired an M2-like antinociceptive phenotype. Indeed, neuropathic allodynia was attenuated after conditional knockout of miR-21 and restored by TGF-βR inhibitor (SB431542) administration. Since TGF-βR2 and TGF-β1 are known miR-21 targets, we suggest that miR-21 transfer from injured neurons to macrophages maintains a proinflammatory phenotype via suppression of such an antiinflammatory pathway. These data support miR-21 inhibition as a possible approach to maintain polarization of DRG macrophages at an M2-like state and attenuate neuropathic pain.

## Graphical abstract

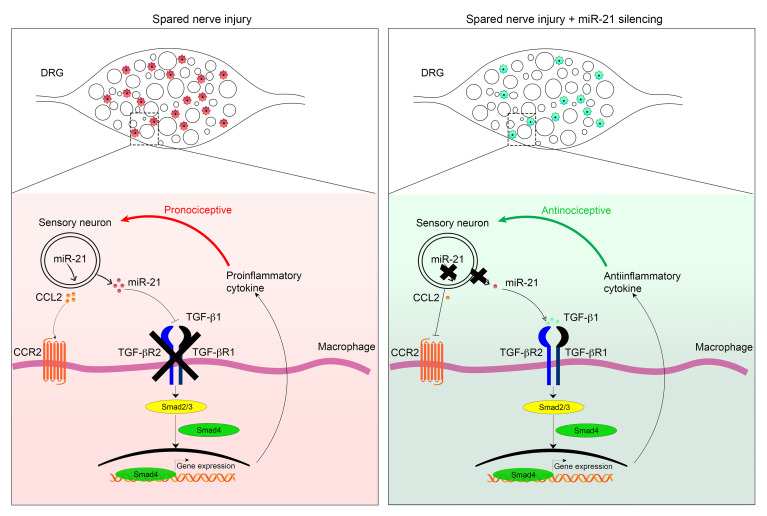

## Introduction

Peripheral neuropathy is a debilitating condition that often leads to severe and chronic neuropathic pain, for which novel treatment strategies are needed because current drugs have limited efficacy and severe side effects (1, 2). In neuropathic pain states, neuronal activity at the site of nerve injury, in dorsal root ganglia (DRGs), and the dorsal horn of the spinal cord provokes immune system responses. Thus, central and peripheral sensitizations are facilitated by microglia, the resident immune cells of the CNS, and monocytes/macrophages in the PNS, respectively (3, 4).

Macrophages are a diverse cell population that exhibits remarkable plasticity after injury and disease and expresses specific hallmarks of their tissue of residence (5–7). Besides a primary function in host defense and inflammatory response, macrophages can release mediators, such as cytokines, that sensitize sensory neurons and contribute to neuropathic pain mechanisms (8). Specifically, after peripheral nerve injury, sensory neuron–associated macrophages (sNAMs) accumulate in lumbar DRGs and at the site of nerve injury (9–11), and play a mechanistic role, as monocyte/macrophage depletion prevents neuropathic pain-like behavior (12, 13). In response to peripheral axon injury, sensory neuron cell bodies upregulate chemokines such as CCL2, which promotes infiltration of monocytes/macrophages in DRGs (14, 15) through the satellite cell sheath around the primary sensory neuron in an attempt to clear damaged neurons (16, 17). Hence, blood-derived macrophages engraft the pool of sNAMs, rapidly skew to a proinflammatory M1-like phenotype, and facilitate mechanisms of chronic pain (16). Conversely, M2-like macrophages play an antinociceptive role in the resolution of chronic pain, as for instance they can transfer mitochondria contained in vesicles to sensory neurons, which have a high metabolic demand under inflammatory pain conditions (18).

Therefore, the definition of specific modalities that underlie DRG neuron-macrophage communication in initiation, maintenance, and resolution of neuropathic pain holds considerable promise toward the identification of targets for novel antinociceptive strategies.

In this study, we focused on a DRG pathway that includes the upregulation of miR-21 in nociceptive neurons after peripheral injury, and neuron-mediated transfer of exosomes containing miR-21 to macrophages to promote a proinflammatory M1-like phenotype (16). Such a neuron-derived miR-21 contributes to neuropathic pain mechanisms, as both conditional knockout of miR-21 in sensory neurons (miR-21 cKO) and intrathecal delivery of a miR-21 antagomir (antagomir-21) attenuate the development of allodynia (16, 19–21). Notably, DRG macrophages isolated from miR-21–cKO mice show significant alteration of miR-21 predicted targets 7 days after peripheral nerve injury (spared nerve injury, SNI) (16). Since a single microRNA (miRNA) can address a multitude of genetic and epigenetic changes, here we identified miR-21 posttranscriptional targets in macrophages following SNI. Our rationale is that the identification of miR-21–mediated mechanisms that regulate macrophage polarization toward a pronociceptive phenotype can provide elements that can be targeted in macrophages, for instance through DRG-specific delivery of nanoparticles.

## Results

### miR-21 induces a specific gene expression profile in macrophages.

Further analysis of our genome-wide microarray in WT and miR-21–cKO DRG macrophages (16) revealed dysregulation of genes associated with cell-cell communication, GPCR ligand binding, and TGF-β signaling. Specifically, *Tgfbr2* (the gene coding for TGF-β receptor 2, TGF-βR2), was upregulated in cKO macrophages compared with WT (*Tgfbr2*: *P* = 0.05, 3.02-fold increase) alongside *Tgfbr3* (coreceptor of *Tgfbr2*), *Tgfb1* (ligand for both receptors), and *Nfya* (encoding nuclear transcription factor Y), which regulates *Tgfbr2* transcription (22) (Supplemental Figure 1A; supplemental material available online with this article; https://doi.org/10.1172/JCI164472DS1). Such gene changes in DRG macrophages were strengthened by results obtained in peritoneal macrophages (PMs) transfected with antagomir-21 to downregulate endogenous expression of miR-21, and then exposed to sensory neuron–derived exosomes overexpressing miR-21 to promote transfer of miR-21 from neurons to macrophages. We found 816 differentially expressed genes (DEGs) compared with antagomir-21–transfected PMs, which themselves displayed 4,979 genes that were differentially regulated compared with scramble-transfected PMs (Supplemental Figure 1, B and C). Since macrophages exposed to neuronal exosomes overexpressing miR-21 showed 2,922 DEGs compared with antagomir-21–treated PMs (Supplemental Figure 1, B and C), these data suggest that neuron-derived miR-21 modulates the transcriptional profile in macrophages in addition to endogenous miR-21. Macrophage exposure to neuronal exosomes affected pathways related to innate immune system responses and cellular responses to stress and metabolism (Supplemental Figure 1D). Indeed, we observed upregulation of *Tnfrsf19* (member of the TNF receptor superfamily; *P* = 0.029, 1.46-fold increase), together with downregulation of *Mrc1* (*P* = 0.036, 1.69-fold decrease) (Supplemental Figure 1E), all of which are associated with a proinflammatory macrophage phenotype. Furthermore, silencing miR-21 expression in macrophages increased the expression of genes associated with cellular responses to TGF-β. For instance, we observed that *Tgfbr1* and *Tgfbr2* were upregulated in PMs lacking miR-21 and gene expression returned to basal levels when miR-21–silenced macrophages were incubated with neuron-derived miR-21 (Supplemental Figure 1F). These data suggest that uptake of neuron-derived miR-21 by macrophages results in alteration of the gene expression profile and pathways that are normally under miR-21 control, such as the TGF-β pathway. To validate our bioinformatics analyses, we evaluated the primary macrophage phenotype after transfection with antagomir-21 and a miR-21 mimic (mimic-21).

### miR-21 fosters a partial proinflammatory phenotype in macrophages.

We began by assessing miR-21 expression in PMs, bone marrow–derived macrophages (BMDMs), and DRG macrophages and found that cultured cells expressed comparable levels that were 28-fold higher than the ex vivo DRG macrophage content (Table 1). Yet, we detected higher levels of macrophages in the ipsilateral compared with contralateral DRG (Table 1).

Thus, in PMs and BMDMs we manipulated miR-21 expression using mimic-21 and antagomir-21 transfection, and quantified expression of MHCII and CD206 using flow cytometry.

**Figure 1:**
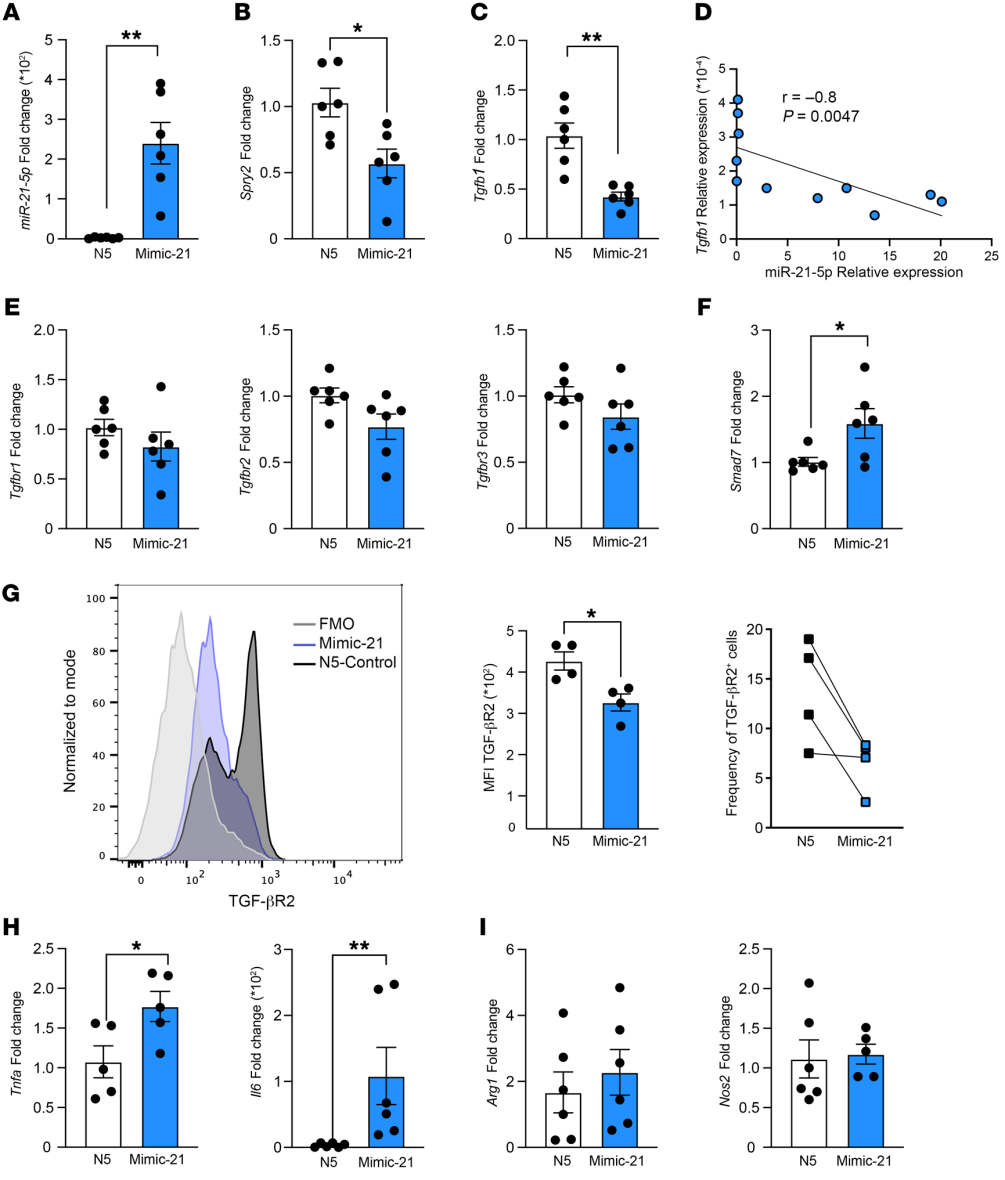
miR-21 induces a proinflammatory phenotype and downregulates TGF-β–related pathway in macrophages. Peritoneal macrophage (PM) transfection with miR-21 mimic (mimic-21) or scramble control (N5) followed by RT-qPCR and flow cytometry. (**A**) miR-21-5p fold change after 48 hours of transfection with mimic-21 (*n* = 6). (**B**) *Spry2* (known target of miR-21-5p) mRNA fold change after 48-hour PM transfection (*n* = 6). (**C**) *Tgfb1* fold change in PMs overexpressing miR-21-5p, *n* = 6 per group. (**D**) Spearman’s correlation between miR-21-5p expression and *Tgfb1* mRNA expression (*n* = 11). (**E**) RT-qPCR of *Tgfbr1*, *Tgfbr2*, *Tgfbr3*, and (**F**) *Smad7* fold change in PMs overexpressing miR-21-5p, *n* = 6 per group. (**G**) Histograms of TGF-βR2 expression in BMDMs transfected with mimic-21 or scramble N5 by quantitative flow cytometry using fluorescence minus one (FMO) controls. The bar graphs represent the MFI (left) and percentage of cells (right), *n* = 4 per group. (**H**) RT-qPCR of proinflammatory genes *Tnfa* and *Il6* in PMs transfected with mimic-21 (*n* = 6). (**I**) RT-qPCR of polarization markers *Arg1* and *Nos2* in PMs transfected with mimic-21, *n* = 5–6 per group. Data are presented as mean ± SEM. *P < 0.05, **P < 0.01 by unpaired, 2-tailed Student’s *t* test (**A**–**C** and **E**–**I**).

PM transfection with mimic-21 resulted in a 240-fold increase in miR-21-5p, but not miR-155 and miR-706 expression, and a decrease in the miR-21 target Spry2 compared with scramble (N5-control) (Figure 1, A and B, and Supplemental Figure 2, A and B), indicating high efficiency of transfection. In mimic-21– compared with scramble-transfected macrophages, we found a decrease in *Tgfb1* transcripts and a negative Spearman’s correlation (*r* = –0.8, *P* = 0.0047) between *Tgfb1* and miR-21-5p expression (Figure 1, C and D), but *Tgfbr1*, *Tgfbr2*, and *Tgfbr3* transcripts were not changed (Figure 1E). We also observed upregulation of *Smad7*, which encodes an endogenous inhibitory R-SMAD involved in the TGF-β signaling pathway (23) (Figure 1F), but no change in *Smurf2*, *Bmpr2*, *Bmp*, and *Smad5*, which are implicated in the TGF-β signaling pathway (23) (Supplemental Figure 2, C–F). Next, flow cytometry analysis of BMDMs transfected with mimic-21 showed lower expression of TGF-βR2 at the single-cell level (mean fluorescence intensity, MFI) and a lower percentage of TGF-βR2^+^ cells, compared with scramble-transfected BMDMs (Figure 1G) (gating strategy in Supplemental Figure 2G), but no difference in MHCII^+^CD206^–^ (M1-like) and MHCII^–^CD206^+^ (M2-like) populations (Supplemental Figure 2H). However, quantification of M1- and M2-like marker gene expression revealed an increase in *Tnfa* and *Il6* (Figure 1H) but no change in *Arg1*, *Nos2*, *Rela*, and *Ym1* (Figure 1I and Supplemental Figure 2, I and J) in mimic-21– compared with scramble-transfected BMDMs. Therefore, a higher level of miR-21 in primary macrophages induces downregulation of antiinflammatory cytokine *Tgfb1* and *Tgfbr2* and upregulation of proinflammatory cytokine *Tnfa* and *Il6* gene products.

**Figure 2:**
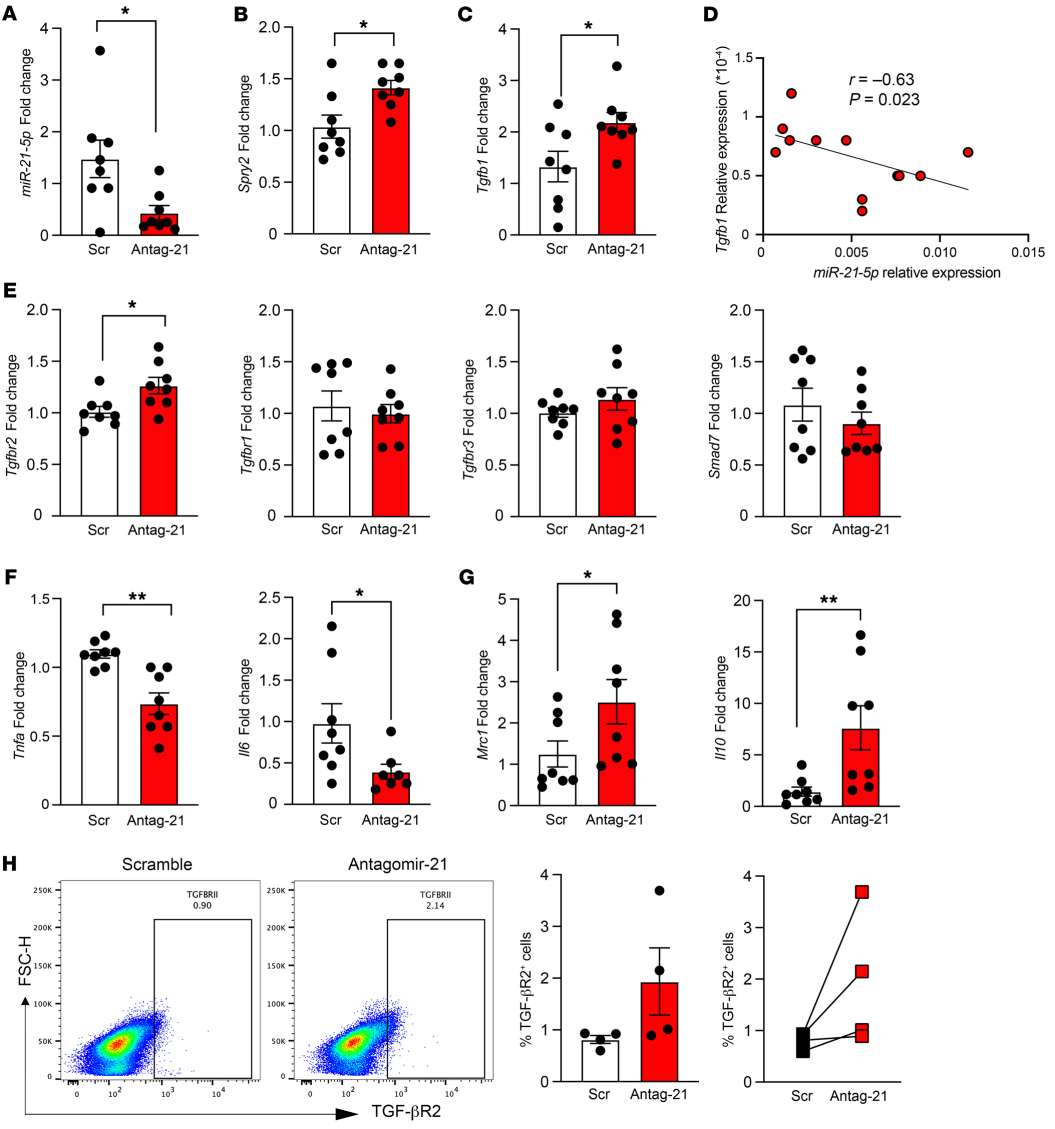
miR-21 silencing induces an antiinflammatory phenotype and upregulates TGF-β–related pathway in macrophages. Macrophages transfected with an miR-21 antagomir (antagomir-21) or scramble control followed by RT-qPCR and flow cytometry. (**A**) miR-21-5p fold change in BMDMs after 48-hour transfection with antagomir-21 (*n* = 8). (**B**) *Spry2* mRNA fold change in BMDMs after 48-hour transfection with antagomir-21 (*n* = 8). (**C**) *Tgfb1* fold change in BMDMs after transfection with antagomir-21, *n* = 8 per group pooled from 2 independent experiments. (**D**) Spearman’s correlation between miR-21-5p expression and *Tgfb1* mRNA expression (*n* = 11). (**E**) RT-qPCR of *Tgfbr2*, *Tgfbr1*, *Tgfbr3*, and *Smad7* in BMDMs after 48-hour transfection with antagomir-21 (*n* = 8). (**F**) RT-qPCR of proinflammatory genes *Tnfa* and *Il6* in BMDMs transfected with antagomir-21 (*n* = 8). (**G**) RT-qPCR of antiinflammatory genes *Mrc1* and *Il10*, *n* = 8 per group, pooled from 2 independent experiments. (**H**) Flow cytometry analysis of TGF-βR2 expression in BMDMs after silencing miR-21-5p, *n* = 4 per group. The bar graphs represent the percentage of F4/80^+^TGF-βR2^+^ cells. Data are presented as mean ± SEM. *P < 0.05, **P < 0.01 by unpaired, 2-tailed Student’s *t* test (**A**–**C** and **E**–**G**).

However, using antagomir-21, we obtained only 50% reduction in miR-21 expression in PMs (Supplemental Figure 3A) and a better yield in BMDMs, with 70% reduction in miR-21 expression in 98% of cells (Figure 2A and Supplemental Figure 3B). Thus, we used antagomir-21–transfected BMDMs and found an increase in *Spry2* and *Tgfb1* (Figure 2, B and C) and significant negative Spearman’s correlation (*r* = –0.63, *P* = 0.0232) between miR-21 and *Tgfb1* expression (Figure 2D). Remarkably, we found an increase in *Tgfbr2* in antagomir-21– compared with scramble-transfected BMDMs, but no changes in *Tgfbr1*, *Tgfbr3*, and *Smad7* (Figure 2E). Moreover, *Tnfa* and *Il6* were decreased and *Mrc1* and *II10* increased (Figure 2, F and G), while other polarization markers, including *Ym1* and *Arg1*, remained unaltered (Supplemental Figure 3D). Flow cytometry analysis of antagomir-21– and scramble-transfected BMDMs showed trends toward upregulation of TGF-βR2 (Figure 2H), an increase in MHCI^–^CD206^+^ (M2-like) cells, and a decrease in MHCII^+^CD206^–^ (M1-like) cells (Supplemental Figure 3, E and F). Therefore, antagomir-21 transfection resulted in a vertically flipped mirror image of mimic-21, as *Tgfb* and *Tgfbr2* were upregulated and *Tnfa* and *Il6* downregulated.

Overall, these data indicate that miR-21-5p fosters a partial proinflammatory phenotype in macrophages. This suggestion is supported by proinflammatory cytokine upregulation in macrophages overexpressing miR-21 and polarization toward an antiinflammatory phenotype in the absence of miR-21. Moreover, BMDMs behave as a faithful surrogate for DRG macrophages and were used in subsequent selected experiments.

### miR-21 regulates TGF-β1 release and SMAD activation in macrophages.

**Figure 3:**
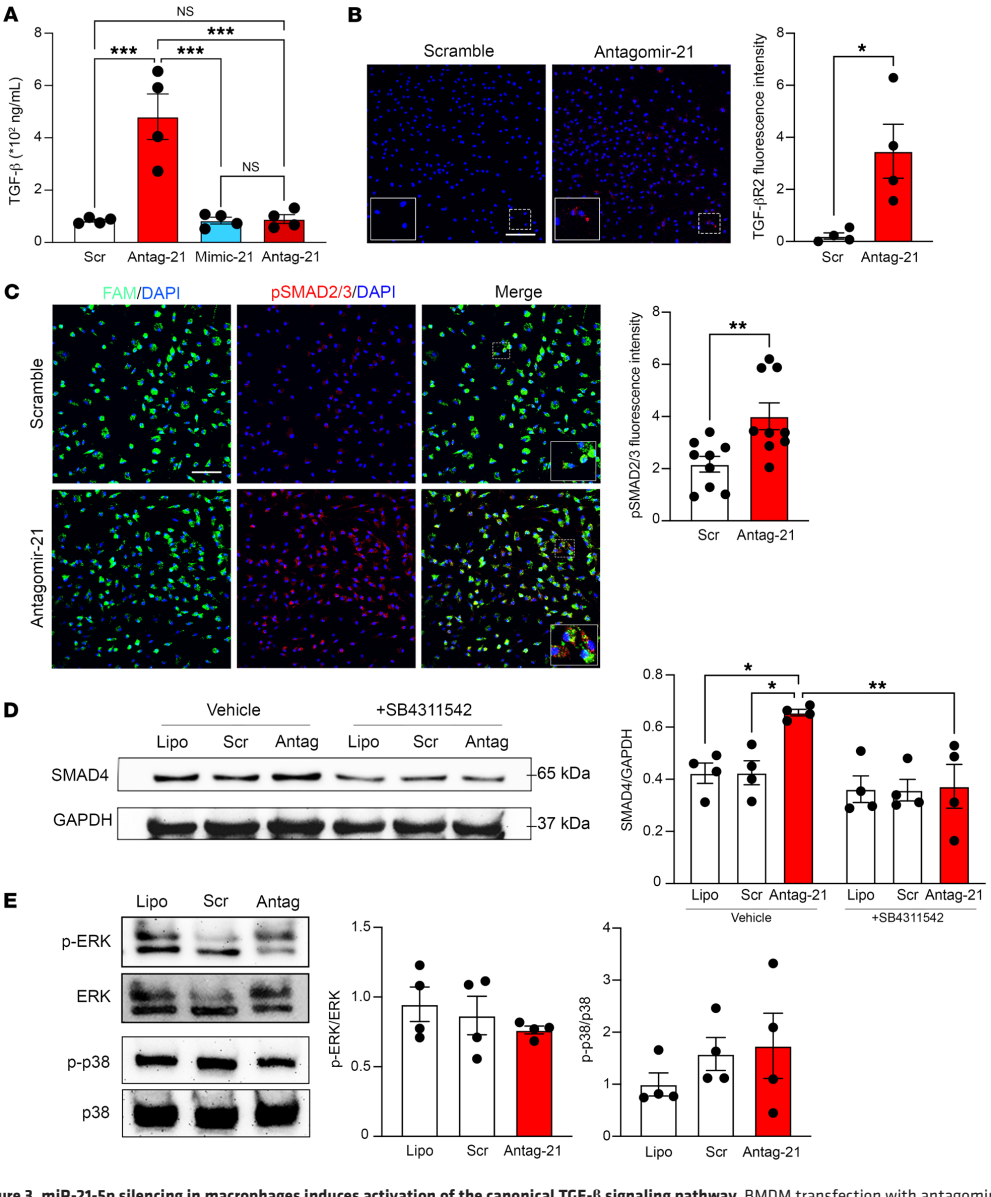
miR-21-5p silencing in macrophages induces activation of the canonical TGF-β signaling pathway. BMDM transfection with antagomir-21 or scramble control for 48 hours followed by TGF-β signaling pathway analysis. (**A**) TGF-β1 ELISA in culture media of BMDMs transfected with antagomir-21, mimic-21, or scramble control, stimulated with vehicle or LPS (100 ng/mL), *n* = 4 per group. (**B**) Immunofluorescent staining of TGF-βR2 in BMDMs transfected with antagomir-21 or scramble control. Bar graph represents quantification of TGF-βR2 fluorescence intensity (*n* = 4). Scale bar: 50 μm. (**C**) Immunofluorescent staining of p-SMAD2/3 in BMDMs transfected with antagomir-21 or scramble control. Bar graph represents quantification of p-SMAD2/3 fluorescence intensity (*n* = 9). Scale bar: 50 μm. (**D**) Western blotting of SMAD4 in BMDMs not transfected (lipofectamine, Lipo) and transfected with either antagomir-21 or scramble control, treated with vehicle or TGF-βR1 antagonist (SB431542), *n* = 4 per group. (**E**) Western blotting for p-ERK/ERK and p-p38/p38 in BMDMs not transfected and transfected with either antagomir-21 or scramble control, *n* = 4 per group. Data are presented as mean ± SEM. *P < 0.05; **P < 0.01; ***P < 0.001 by 1-way ANOVA followed by Tukey’s multiple-comparison test (**A** and **D**) or unpaired, 2-tailed Student’s *t* test (**B** and **C**).

In antagomir-21–transfected BMDMs, consistent with the *Tgfb1* mRNA increase, we found higher TGF-β1 extracellular levels than in scramble-transfected BMDMs (Figure 3A). Such a release of TGF-β1 was lowered to control levels by incubation with LPS, which itself upregulates miR-21 by 8.57 ± 0.997-fold, (*n* = 4, *P* < 0.05) and likely replenished miR-21 in antagomir-21–transfected BMDMs. In addition, TGF-βR2 immunostaining was higher in antagomir-21– than scramble-transfected BMDMs (Figure 3B), along with more SMAD2/3 phosphorylation, and a significant upregulation of SMAD4 that was blocked by the presence of SB431542, a TGF-βR1 inhibitor (Figure 3, C and D). The choice of the TGF-βR1 antagonist was due to TGF-βR1 being the signaling protein required for formation of the active complex with TGF-βR2, which acts as the initial binding protein for TGF-β1 (23). These data suggest that lack of miR-21 in macrophages results in higher basal release of TGF-β1 and activation of TGF-βR2 followed by TGF-βR1–mediated signaling through a SMAD-dependent pathway. This possibility is reinforced by the observation that neither ERK nor p38 phosphorylation was altered by antagomir-21 transfection and involvement of a non-SMAD pathway could be ruled out (Figure 3E).

### Sensory neuron–derived miR-21 regulates TGF-β–related pathway in macrophages.

**Figure 4:**
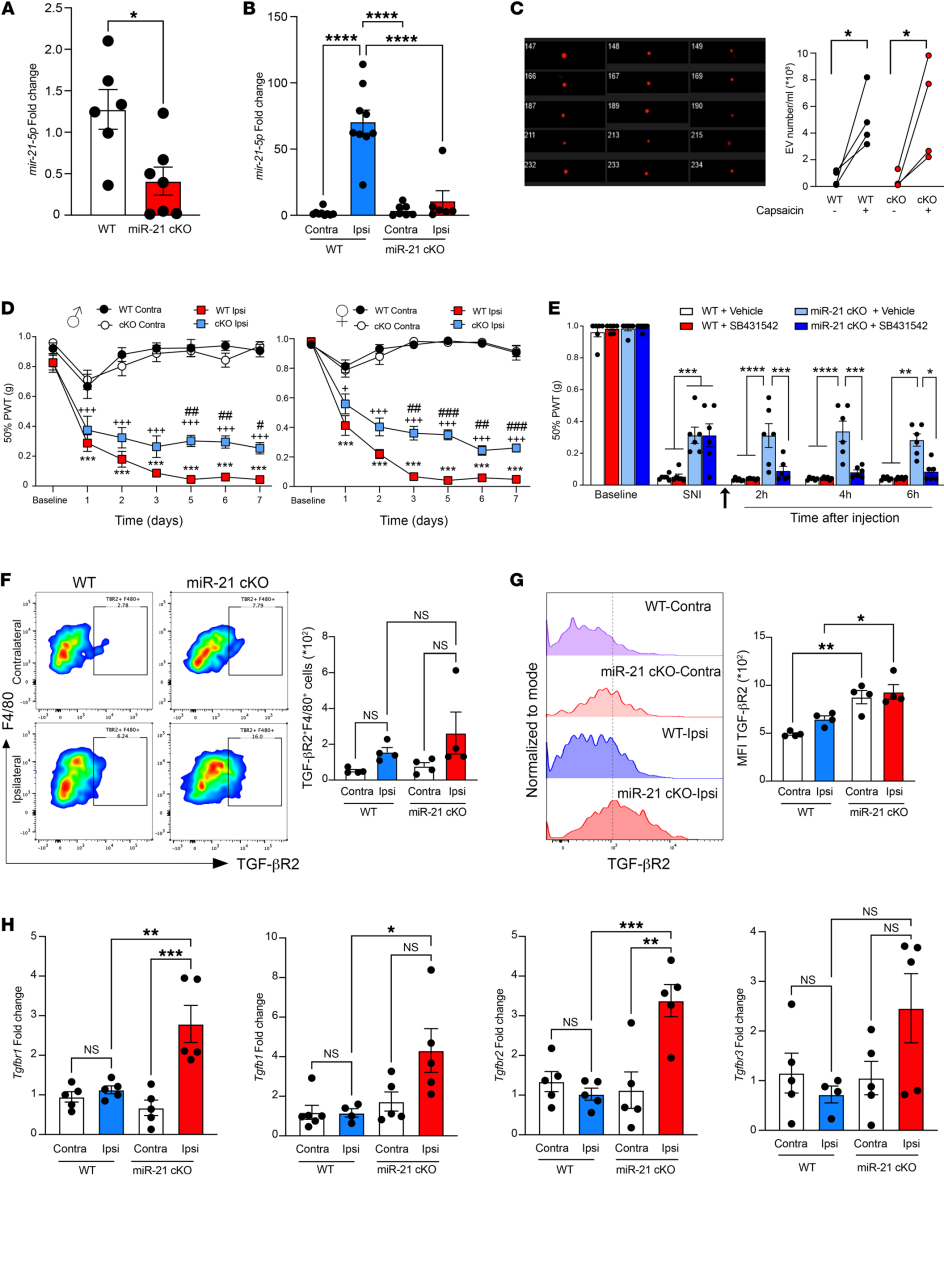
miR-21 silencing in sensory neurons prevents neuropathic hypersensitivity and regulates TGF-βR2 expression. (**A**) RT-qPCR of miR-21-5p in DRG cultures of WT and miR-21–cKO mice, *n* = 6–7 cultures per group. (**B**) RT-qPCR of miR-21-5p in DRGs of WT and miR-21–cKO mice on day 7 after SNI (*n* = 6–9). (**C**) ImageStream analyses of exosomes (extracellular vesicles, EVs) isolated from culture media of WT and miR-21–cKO DRG neurons incubated with vehicle or capsaicin (CAPS, 1 μM) for 3 hours (*n* = 4). (**D**) Attenuated allodynia in miR-21–cKO mice up to day 7 after SNI in males and females. Data are presented as 50% paw withdrawal thresholds (PWT). ^+^*P* < 0.05, ^+++^*P* < 0.001 compared with miR-21–cKO contralateral thresholds; ***P < 0.001 compared with WT contralateral thresholds; ^#^*P* < 0.05, ^##^*P* < 0.01, ^###^*P* < 0.001 compared with WT ipsilateral thresholds; by 2-way ANOVA followed by Tukey’s multiple-comparison test (*n* = 10–14 per group). (**E**) Intrathecal injection of TGF-βR1 inhibitor SB431542 (100 pmol/mouse) abolished the anti-allodynic effect in miR-21 cKO. Arrow indicates the time of injection given on day 7 after SNI, *n* = 6. (**F**) Representative scatterplots of DRG CD11b^+^F4/80^+^ cells stained for TGF-βR2 on day 7 after SNI. Bar graphs represent TGF-βR2^+^ cell number (*n* = 4). (**G**) Representative histograms of TGF-βR2 expression in DRG CD11b^+^F4/80^+^ cells on day 7 after SNI (MFI), *n* = 4. (**H**) RT-qPCR of *Tgfbr1*, *Tgfb1, Tgfbr2*, and *Tgfbr3* in F4/80^+^ cells isolated from DRGs of WT and miR-21–cKO mice on day 7 after SNI, *n* = 5 independent experiments from 4–6 pooled animals in each. Data are presented as mean ± SEM. *P < 0.05; **P < 0.01; ***P < 0.001; ****P < 0.0001 by unpaired, 2-tailed Student’s *t* test (**A**) or 1-way ANOVA followed by Tukey’s multiple-comparison test (**B**, **C**, and **E**–**H**).

Our next step was to move to an in vivo setting and validate the hypothesis that sensory neuron–derived miR-21 contributes to the development of neuropathic allodynia through downregulation of the TGF-β–mediated pathway in DRG macrophages. For this purpose, we used our miR-21–cKO mice that show significant reduction of miR-21 expression in DRG compared with WT (Figure 4A), and lack of miR-21 upregulation in DRG ipsilateral to nerve injury (Figure 4B). Furthermore, since neurons release miR-21 encapsulated in exosomes, we confirmed that exosome release under basal conditions and following activation of nociceptors by capsaicin was not altered in miR-21–cKO mice (Figure 4C). Then, in behavioral studies, we confirmed that male and female cKO mice developed less severe neuropathic allodynia than WT at 5 to 7 days after SNI (Figure 4D). Additionally, we observed that single intrathecal administration of SB431542 restored allodynia in miR-21–cKO but not WT mice at 2, 4, and 24 hours after injection (Figure 4E), suggesting that endogenous TGF-β1 exerted antinociceptive effects in miR-21–cKO mice. Consistent with this possibility, intrathecal infusion of TGF-β1 reduces neuropathic allodynia by about 30% (24), which is comparable to the attenuation of neuropathic allodynia we observed in miR-21 cKO. Moreover, considering that we used SB431542 at a dose of 100 pmol/mouse that completely blocks the TGF-β1–induced anti-allodynic effect in neuropathic mice (25), these results suggest that, in miR-21–cKO mice, the attenuation of allodynia requires TGF-βR1 activation.

In keeping with the possibility that TGF-β pathway–related changes occur in macrophages 7 days after SNI, F4/80^+^ cells from miR-21–cKO ipsilateral DRGs showed a trend toward higher TGF-βR2^+^ events and substantially higher TGF-βR2 expression at the cellular level compared with WT (Figure 4, F and G). Moreover, miR-21–cKO isolated F4/80^+^ cells displayed upregulation of *Tgfbr1* and *Tgfbr2,* and a trend toward *Tgfb1* upregulation, but no change in *Tgfbr3* compared with WT (Figure 4H). In contrast, in the non-leukocyte CD45^–^ fraction of the DRG, *Tgfb1*, *Tgfbr1*, *Tgfbr2*, and *Tgfbr3* expression was unaltered (Supplemental Figure 4A), suggesting that miR-21 does not regulate the TGF-β pathway in neurons. Yet, in macrophages such TGF-β–related variations occurred concomitant with upregulation of antiinflammatory markers *Mrc1* and *Il10* (Supplemental Figure 4B) and no change in the proinflammatory markers *Il6* and *Tnfa* (Supplemental Figure 4C). Contralateral DRG macrophages were used for quantification of macrophages under control conditions, since cKO contralateral thresholds were comparable to both WT contralateral and sham thresholds (Supplemental Figure 4D).

Overall, these data suggest that in DRGs the absence of neuron-derived miR-21 results in activation of a TGF-β pathway and M2-like polarization of macrophages. These results correlate with both attenuation of neuropathic allodynia in miR-21–cKO mice and restoration of allodynia following injection of a TGF-βR inhibitor.

### Neuronal miR-21 regulates classical CCR2 monocyte/macrophage infiltration in DRGs.

**Figure 5:**
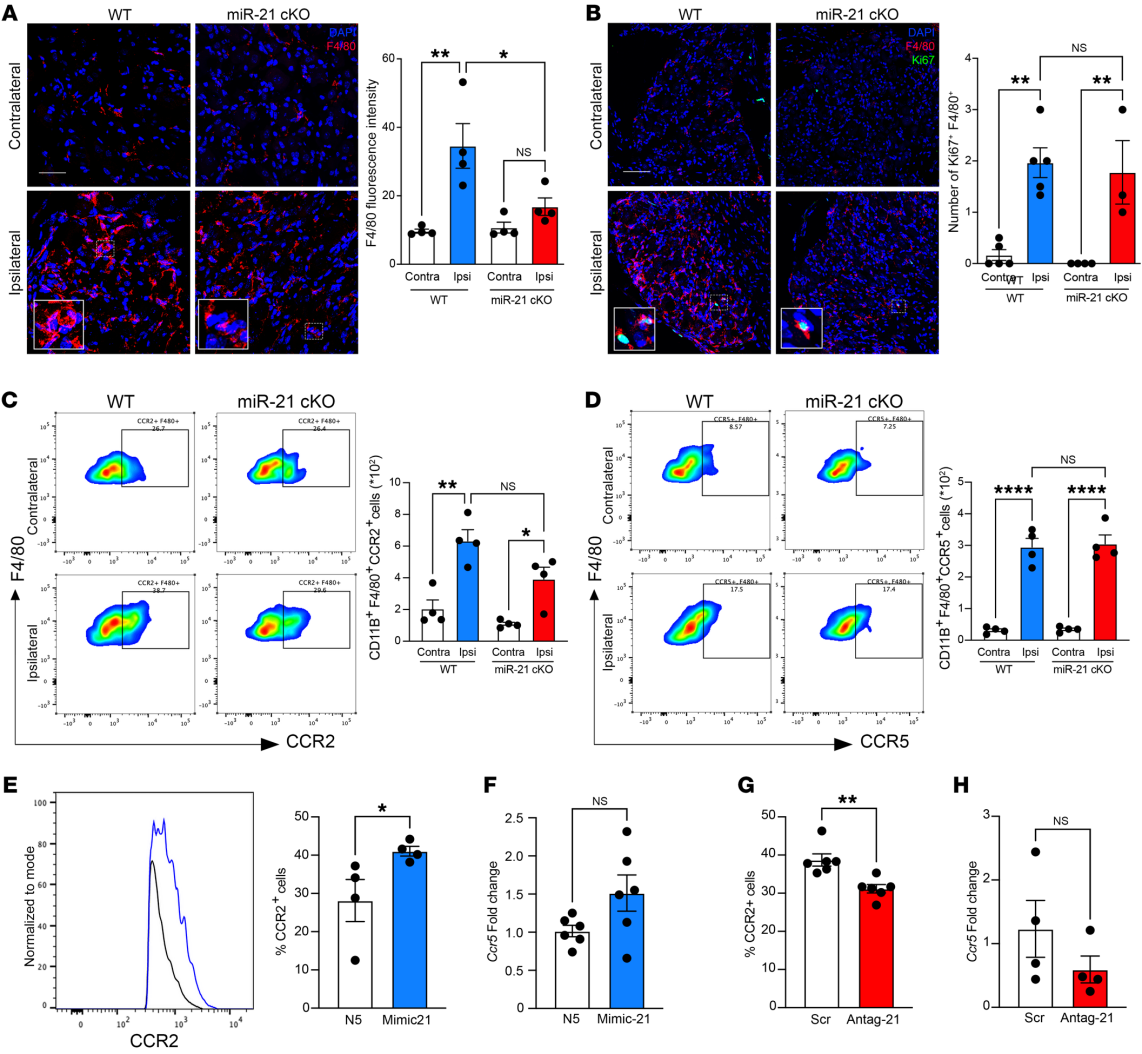
miR-21 silencing in sensory neurons reduces CCR2^+^ monocyte/macrophage infiltration. (**A**) Immunofluorescent staining of F4/80 in ipsilateral and contralateral DRGs of WT and miR-21–cKO mice on day 7 after SNI. Scale bar: 20 μm (*n* = 4, repeated 3 times). (**B**) Immunostaining of F4/80 and Ki67 in ipsilateral and contralateral DRGs of WT and miR-21–cKO mice on day 7 after SNI. Scale bar: 20 μm (*n* = 4). (**C**) Representative scatterplots of CCR2 expression in DRG CD11b^+^F4/80^+^ cells of WT and miR-21–cKO mice on day 7 after SNI (*n* = 4); the bar graph represents CCR2^+^ absolute cell number. (**D**) Representative scatterplots of CCR5 expression in DRG CD11b^+^F4/80^+^ cells of WT and miR-21–cKO mice on day 7 after SNI (*n* = 4); the bar graph represents CCR5^+^ absolute cell number. (**E**) Flow cytometry analysis of CCR2 expression in PMs transfected with N5 control or mimic-21 (*n* = 4). The blue line corresponds to mimic-21 and the black to N5-scramble control. (**F**) RT-qPCR of *Ccr5* in PMs transfected with N5 control or mimic-21, *n* = 8. (**G**) Flow cytometry analysis of CCR2 expression in BMDMs transfected with scramble control or antagomir-21 (*n* = 6). (**H**) RT-qPCR of *Ccr5* in BMDMs transfected with scramble control or antagomir-21 (*n* = 4). Data are presented as mean ± SEM. *P < 0.05; **P < 0.01; ****P < 0.0001 by 1-way ANOVA followed by Tukey’s multiple-comparison test (**A**–**D**) or unpaired, 2-tailed Student’s *t* test (**E**–**H**).

Having confirmed our published data (16) showing that F4/80^+^ cell accumulation is higher in ipsilateral than contralateral WT DRGs after nerve injury, while it is lower in ipsilateral miR-21–cKO DRGs than WT (Figure 5A), we next evaluated whether neuronal miR-21 affected macrophage proliferation and infiltration on day 7 after SNI. We observed that both WT and cKO mice showed more Ki67^+^F4/80^+^ macrophages in the ipsilateral DRG than contralateral, suggesting that neuron-derived miR-21 does not affect in situ macrophage proliferation (Figure 5B). Concerning potential effects on infiltration in DRGs after SNI, we monitored expression of 2 chemokine receptors, CCR2 and CCR5, which are implicated in monocyte/macrophage infiltration (26, 27). In flow cytometry analysis of CD11b^+^F4/80^+^ macrophages of WT DRGs, we found higher numbers of both CCR2^+^ and CCR5^+^ cells in ipsilateral compared with contralateral DRGs (Figure 5, C and D). However, in cKO DRGs, CCR2^+^ cell number was lower than in WT (Figure 5C), while CCR5^+^ cell accumulation was unchanged (Figure 5D), suggesting that miR-21 affected CCR2^+^ cell infiltration in DRGs. Notably, these observations were all specific to DRG macrophages, as we did not observe changes in either macrophage infiltration or CCR2 expression at the site of nerve injury (Supplemental Figure 5A). These data in DRG macrophages were further substantiated in transfection experiments, as we observed higher numbers of CCR2^+^ cells (Figure 5E) and a trend toward upregulation of *Ccr5* mRNA (~40%; Figure 5F) in mimic-21– compared with scramble-transfected BMDMs. Conversely, in antagomir-21–transfected BMDMs we found lower numbers of CCR2^+^ cells (Figure 5G) and reduced, yet not significantly, *Ccr5* expression (Figure 5H).

**Figure 6:**
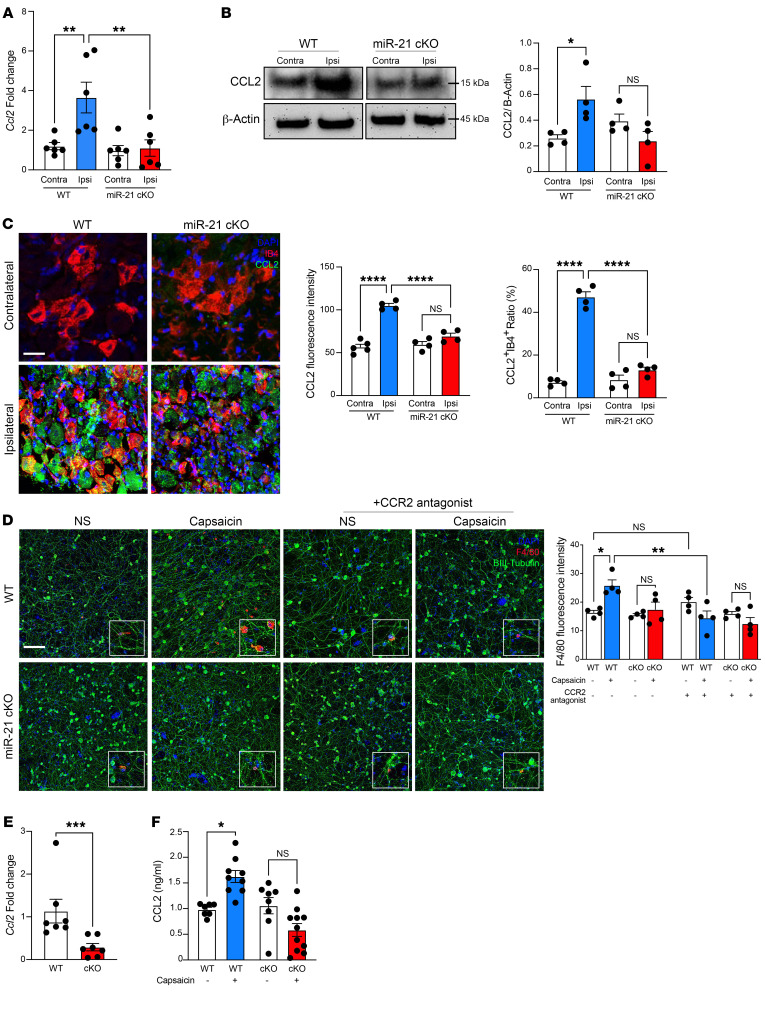
miR-21 silencing reduces CCL2 in sensory neurons and blocks macrophage transmigration. (**A**) RT-qPCR of *Ccl2* in ipsilateral and contralateral DRGs from WT and miR-21–cKO mice on day 7 after SNI, *n* = 6. (**B**) Western blotting of CCL2 in ipsilateral and contralateral DRGs from WT and miR-21–cKO mice on day 7 after SNI, *n* = 4. (**C**) Immunofluorescent staining of CCL2 (green) and IB4 (red) in ipsilateral and contralateral DRGs of WT and miR-21–cKO mice on day 7 after SNI, *n* = 4. Scale bar: 10 μm. (**D**) Representative Transwell photomicrograph of WT and miR-21–cKO DRG neurons stimulated with vehicle or capsaicin for 3 hours, stained for βIII-tubulin (green), and BMDMs treated with vehicle or CCR2 antagonist, stained for F4/80 (red), *n* = 4. Scale bar: 20 μm. (**E**) RT-qPCR of *Ccl2* in WT and miR-21–cKO DRG culture (*n* = 7). (**F**) CCL2 ELISA in culture media of DRG neurons of WT and miR-21–cKO mice, treated with vehicle or capsaicin, *n* = 7–11. Data are presented as mean ± SEM. *P < 0.05, **P < 0.01, ***P < 0.001, ****P < 0.0001 by 1-way ANOVA followed by Tukey’s multiple-comparison test (**A**–**D** and **F**) or 2-tailed Student’s *t* test (**E**).

These data suggest that after SNI, miR-21 that is upregulated in neurons can influence CCR2^+^ monocyte/macrophage infiltration in DRGs. We reasoned that for such a mechanism to be relevant, miR-21 might be involved in upregulation of CCL2 in neurons, which occurs in response to axonal injury (28), albeit through an indirect mechanism, as there is no evidence that this chemokine is a miR-21 target. Indeed, we observed that, in the CD45^–^ fraction of the DRG, *Ccl2* mRNA was upregulated in WT, but not miR-21–cKO mice (Figure 6A). In addition, CCL2 protein was increased in WT but not miR-21–cKO ipsilateral compared with contralateral DRG (Figure 6B) and CCL2 immunostaining was higher in ipsilateral than contralateral and especially in IB4^+^ neurons as expected (15, 29), but not in cKO neurons (Figure 6C). Consistently with an alteration of the CCL2 levels in miR-21 cKO, we made the following 2 sets of observations in a Transwell plate assay in which cultured DRG neurons were challenged with capsaicin to provide an in vitro model of nociceptive neuron stimulation (schematic in Supplemental Figure 5B). The first one was that capsaicin promoted transmigration of macrophages toward the WT but not cKO neuronal compartment, and this effect was blocked by a CCR2 antagonist (Figure 6D). The second one was that miR-21–cKO cultured DRG neurons expressed lower intracellular levels of *Ccl2* compared with WT DRGs (Figure 6E) and released lower extracellular CCL2 after incubation with capsaicin (Figure 6F). Therefore, miR-21 modulation of CCL2 levels in neurons, in synergy with a miR-21–associated increase in CCR2 in macrophages, provides neuroimmune crosstalk by which miR-21 regulates monocyte infiltration in the DRG after nerve injury. In addition, our evidence indicates that in such macrophages, neuron-derived miR-21 inhibits expression of TGF-β1 and TGF-β2 and promotes a proinflammatory phenotype. Taking advantage of such a sharp phenotype, we tested the possibility that antagomir-21–transfected BMDMs exert anti-allodynic effects in neuropathic mice.

### Intrathecal delivery of antagomir-21–treated macrophages reverses neuropathic allodynia.

Given the contribution of DRG macrophages to both initiation and maintenance of neuropathic hypersensitivity (12), we tested the effect of BMDMs transfected with antagomir-21 on neuropathic allodynia in SNI, and used GFP^+^ BMDMs polarized with IL-4 and TGF-β (M2-like) for comparison (Supplemental Figure 6A).

**Figure 7:**
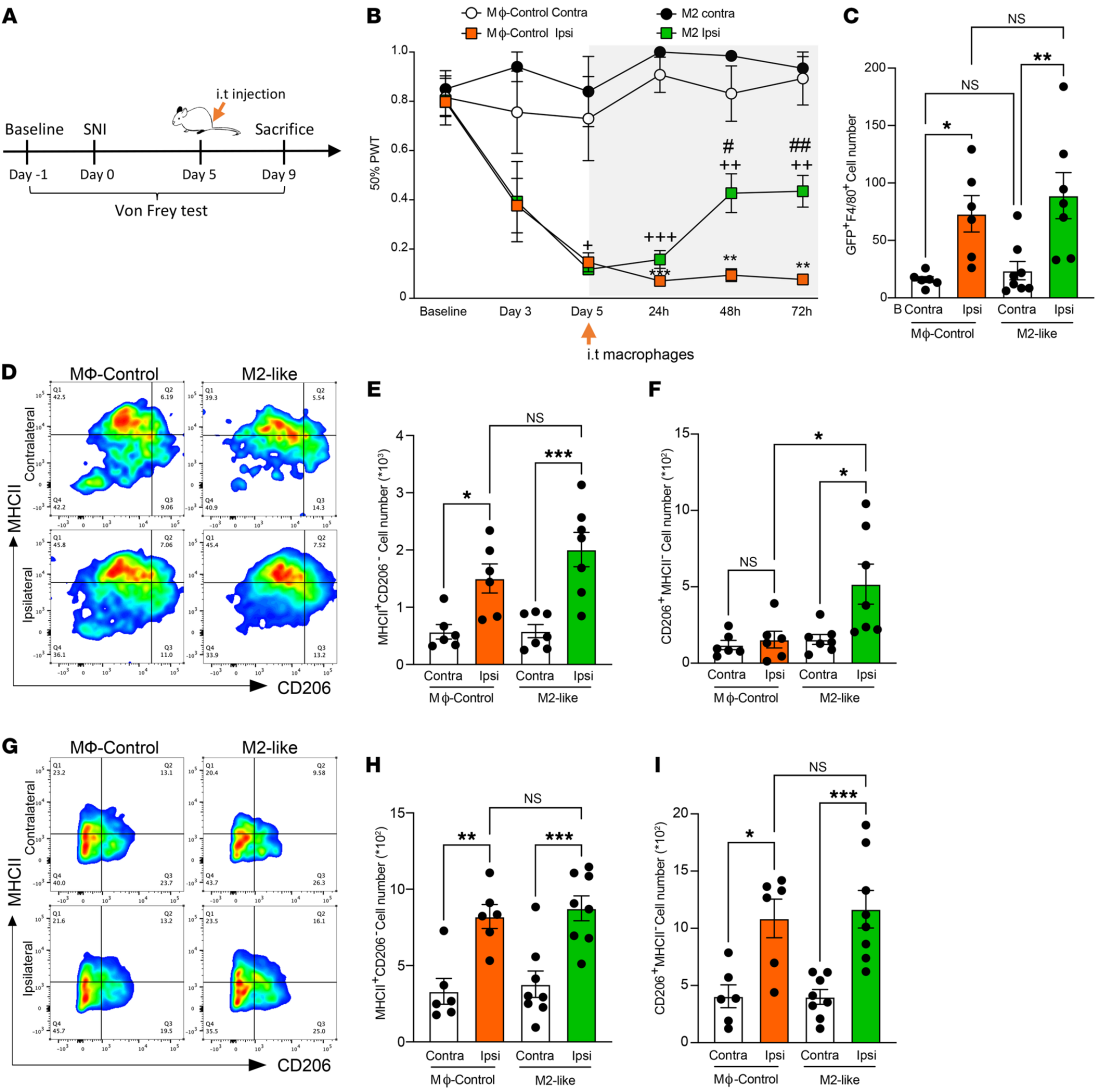
Intrathecal injection of M2-like macrophages alleviates neuropathic allodynia via the polarization of sNAMs toward an antiinflammatory phenotype. (**A**) Schematic representation of the experimental design for intrathecal (i.t.) delivery of macrophages in WT mice, and the behavioral tests. (**B**) Effect of macrophage control (MΦ-control) and M2-like macrophages on the development of mechanical hypersensitivity in SNI (*n* = 5–6). Data are presented as 50% paw withdrawal thresholds (PWT); mean ± SEM. ^+^*P* < 0.05, ^++^*P* < 0.01, ^+++^*P* < 0.001 compared with M2-like contralateral thresholds; **P < 0.01, ***P < 0.001 compared with MΦ-control contralateral thresholds, ^#^*P* < 0.05, ^##^*P* < 0.01 compared with MΦ-control ipsilateral thresholds; by 2-way ANOVA followed by Tukey’s multiple-comparison test. (**C**) Absolute number of GFP^+^F4/80^+^ cells in DRGs 2 hours after i.t. delivery of MΦ-control and M2-like macrophages (*n* = 6–8). (**D**) Representative scatterplots of CD206 and MHCII expression in CD11b^+^F4/80^+^ macrophages in DRGs 2 hours after i.t. delivery of MΦ-control and M2-like macrophages. (**E**) MHCII^+^CD206^–^ (M1-like), and (**F**) CD206^+^MHCII^–^ (M2-like) absolute cell number in DRGs 2 hours after i.t. delivery of MΦ-control and M2-like macrophages (*n* = 6–7). (**G**) Representative scatterplots of CD206 and MHCII expression in CD11b^+^F4/80^+^ macrophages in DRGs 48 hours after i.t. delivery of MΦ-control and M2-like macrophages. (**H**) MHCII^+^CD206^–^ (M1-like) and (**I**) CD206^+^MHCII^–^ (M2-like) absolute cell number in DRGs 48 hours after i.t. delivery of MΦ-control and M2-like macrophages (*n* = 6–8). Data are presented as mean ± SEM. *P < 0.05, **P < 0.01, ***P < 0.001 by 1-way ANOVA followed by Tukey’s multiple-comparison test (**C**, **E**, **F**, **H** and **I**).

**Figure 8:**
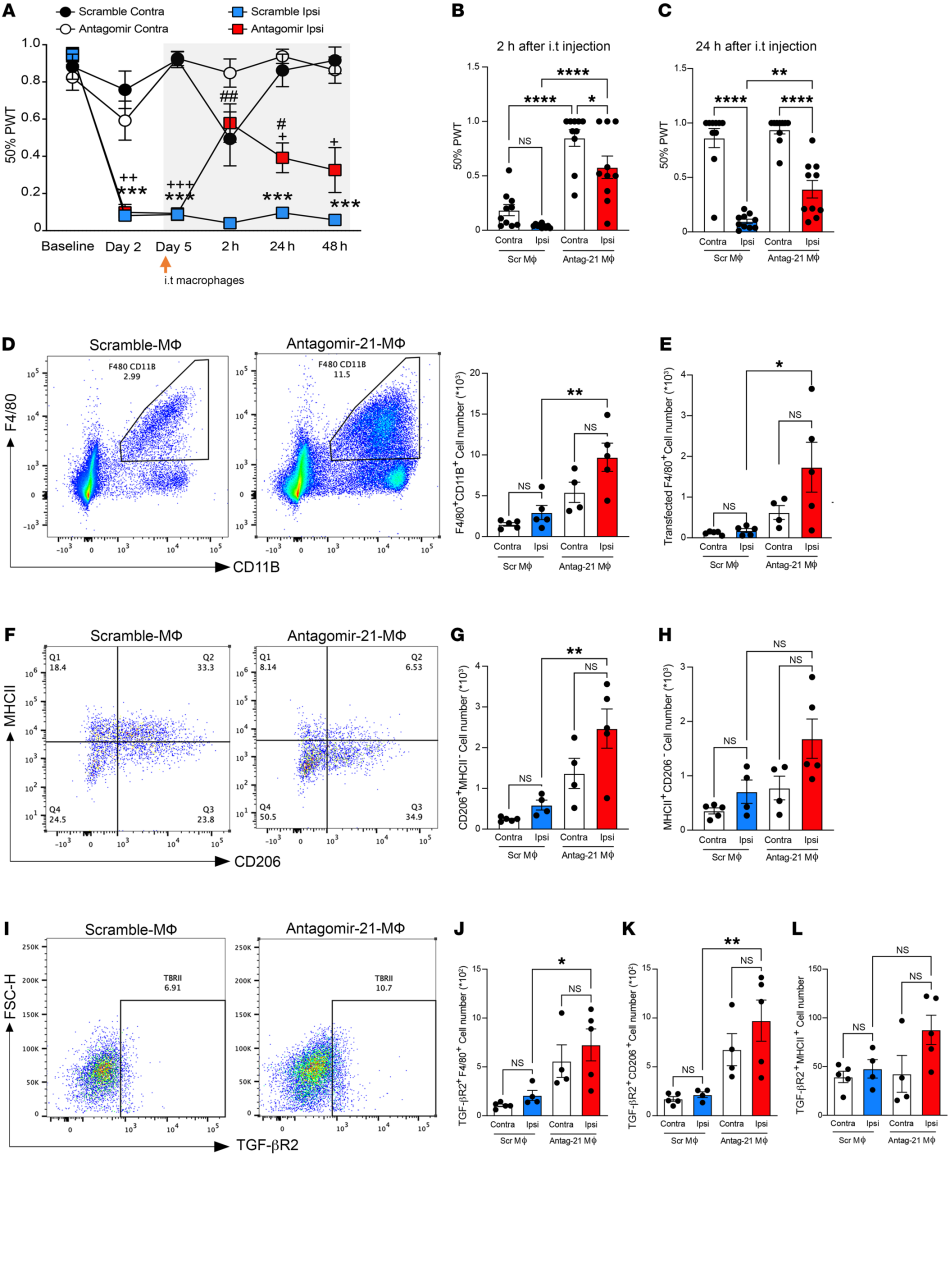
Intrathecal delivery of antagomir-21–treated BMDMs reverses neuropathic hypersensitivity via upregulation of TGF-βR2 at early stages. (**A**) Effect of scramble-treated BMDMs and antagomir-21–treated BMDMs on the development of mechanical hypersensitivity after SNI (*n* = 10). Data are presented as 50% paw withdrawal thresholds (PWT); mean ± SEM. ^+^*P* < 0.05, ^++^*P* < 0.01, ^+++^*P* < 0.001 compared with antagomir-21–treated BMDM contralateral thresholds; ***P < 0.001 compared with scramble-treated BMDM thresholds; ^#^*P* < 0.05, ^##^*P* < 0.01 compared with scramble ipsilateral thresholds; 2-way ANOVA followed by Tukey’s multiple-comparison test. (**B**) Bar graphs represent PWT at 2 hours and (**C**) 24 hours after intrathecal (i.t.) injection of antagomir-21–treated BMDMs or scramble-treated BMDMs (*n* = 10). (**D**) Representative scatterplots of F4/80^+^CD11b^+^ macrophages in ipsilateral DRGs 2 hours after i.t. injection of scramble-treated BMDMs or antagomir-21–treated BMDMs (gated on live cells); the bar graphs represent the F4/80^+^CD11b^+^ absolute cell number (*n* = 4–5). (**E**) Bar graphs of GFP^+^ BMDM absolute cell number in L3-L4-L5 DRGs 2 hours after i.t. injection (*n* = 4–5). (**F**) Representative scatterplots of CD206 and MHCII in CD11b^+^ F4/80^+^ macrophages of ipsilateral DRG 2 hours after i.t. injection. (**G**) Bar graphs represent CD206^+^MHCII^–^ and (**H**) MHCII^+^CD206^–^ absolute cell numbers in CD11b^+^F4/80^+^ macrophages 2 hours after i.t. injection (*n* = 4–5). (**I**) Representative scatterplots of TGF-βR2 expression in CD11b^+^F4/80^+^ macrophages of ipsilateral DRG 2 hours after i.t. injection. (**J**) TGFBR2^+^F4/80^+^ absolute cell numbers (*n* = 4–5). (**K**) TGFBR2^+^CD206^+^ and (**L**) TGFBR2^+^MHCII^+^ absolute cell numbers in the DRG 2 hours after i.t. injection (*n* = 4–5). Data are presented as mean ± SEM. *P < 0.05; **P < 0.01; ***P < 0.001; ****P < 0.0001 by 1-way ANOVA followed by Tukey’s multiple-comparison test (**B**–**E**, **G**, **H**, and **J**–**L**).

For this purpose, we first observed that intrathecal administration of M2-like BMDMs (5 × 10^5^ cells) resulted in 50% to 80% reversal of ipsilateral SNI allodynia at 24, 48, and 72 hours after injection (Figure 7, A and B). Then, using immunofluorescence and flow cytometry we found that intrathecal GFP^+^ BMDMs accumulated mainly in ipsilateral L3-L4-L5 DRGs, and to a lesser extent in contralateral L3-L4-L5 DRGs (Figure 7C), with no detection in lumbar spinal cord (Supplemental Figure 6B), consistent with previous reports (25–30). Furthermore, 2 hours after intrathecal injection of M2-like BMDMs, the ipsilateral pool of DRG macrophages showed the expected increase in MHCII^+^CD206^–^ (M1-like) cells as a result of SNI (Figure 7, D and E), but also higher numbers of CD206^+^MHCII^–^ (M2-like) cells compared with contralateral DRGs (Figure 7, D and F, and Supplemental Figure 6C), although MHCII expression levels were unchanged in the single-cell analysis (Supplemental Figure 6D). Thus, these data indicate that M2-like macrophages differentiated in vitro acquire an antinociceptive phenotype in vivo. Specifically, M2-like BMDMs can engraft the DRG pool of macrophages and maintain an M2-like phenotype, regardless of the presence of an M1-like environment associated with nerve injury. Notably, we observed that at peak reversal of allodynia 48 hours after M2-like BMDM injection, we could still detect higher numbers of CD206^+^MHCII^–^ and MHCII^+^CD206^–^ cells in ipsilateral compared with contralateral DRGs (Figure 7, G–I). In our final set of experiments, we administered antagomir-21–transfected BMDMs (5 × 10^5^) and observed a rapid reversal of ipsilateral neuropathic allodynia compared with scramble-transfected BMDMs (Figure 8A). We detected acute contralateral hypersensitivity after scramble-transfected BMDM injection (Figure 8B), which subsided by 24 hours (Figure 8C). Relevantly, antagomir-21–transfected BMDM reversal of allodynia by approximately 60% is consistent with the 50% to 80% inhibition of SNI allodynia at 24 and 48 hours after intrathecal administration of bone marrow stromal cells (2.5 × 10^5^) that target the DRG and locally secrete TGF-β1 (25). Flow cytometry analysis of ipsilateral CD11b^+^F4/80^+^ cells revealed higher numbers in antagomir-21– compared with scramble-transfected BMDMs (Figure 8D), which correlated with antagomir-21–transfected BMDMs’ tendency to accumulate in DRGs (Figure 8E).

We then investigated expression of CD206 and MHCII markers in the whole DRG macrophage pool, including injected BMDMs and endogenous cells. We observed an increase in the CD206^+^MHCII^–^ population in antagomir-21–BMDM–treated ipsilateral DRGs compared with scramble-BMDMs, with no difference between contralateral DRGs (Figure 8, F and G). However, the MHCII^+^CD206^–^ cell population was not different between antagomir-21–BMDM and scramble-BMDM DRGs (Figure 8, F–H). Therefore, these data show that intrathecal injection of antagomir-21–transfected BMDMs induces reversal of neuropathic allodynia and polarizes DRG macrophages into an M2-like phenotype.

Since we argued that such an M2 polarization shift was unlikely to have occurred within 2 hours of BMDM injection, we further characterized macrophages and found higher numbers of TGF-βR2^+^ cells in both ipsilateral and contralateral DRGs of antagomir-21–BMDM compared with scramble-BMDM injection (Figure 8, I and J). These cells were TGF-βR2^+^CD206^+^ (Figure 8K), whereas TGF-βR2^+^MHCII^+^ cells were very few and remained unaltered (Figure 8L).

In additional analyses 48 hours after BMDM injection, when the anti-allodynic effect had faded slightly, ipsilateral DRG CD11b^+^F4/80^+^ numbers were comparable between antagomir-21– and scramble-BMDM (Supplemental Figure 8A), and similar results were obtained for injected F4/80^+^ BMDMs (Supplemental Figure 8B). Specifically, these cells had not polarized toward either an M1-like or M2-like phenotype (Supplemental Figure 8C), which indicates adaptability of in vitro–differentiated BMDMs to an in vivo environment. Concerning TGF-βR2, we noticed a trend toward upregulation in F4/80^+^ cells in ipsilateral and contralateral DRGs in antagomir-21– compared with scramble-BMDM (Supplemental Figure 8D), a trend that was associated with CD206^+^ but not MHCII^+^ macrophages (Supplemental Figure 8, E and F).

Altogether, these data indicate that intrathecal injection of antagomir-21–transfected BMDMs rapidly alleviates neuropathic allodynia, as macrophages acquire an M2-like phenotype in vivo that is associated with upregulation of TGF-β2R.

## Discussion

This study provides evidence for a dual role of miR-21 in neurons and macrophages that promotes pronociceptive mechanisms and pathways in DRGs following peripheral nerve injury. Specifically, in injured sensory neuron cell bodies, miR-21 contributes to CCL2 upregulation that attracts inflammatory macrophages in DRGs. In macrophages, lack of transfer of sensory neuron–derived miR-21 results in upregulation of TGF-βR2, release of TGF-β1, and fosters an antiinflammatory and antinociceptive phenotype.

Our data bring what we believe is a novel addition to the concept that neurons instruct immune cells through shuttling of miRNA in extracellular vesicles to influence the nature of the immune infiltrate in the DRG, which in turn impacts mechanisms underlying noxious signaling. Thus, miR-21 conditional deletion in sensory neurons opposes de novo expression of the chemokine CCL2, which is normally associated with axonal injury (31, 32), and results in reduction of CCR2-expressing immune cells in DRGs. Since miRNAs inhibit their own target expression and there is no evidence that CCL2 is a miR-21 target, this effect on chemokine expression is likely to be indirect. For instance, following nerve injury, in sensory neurons miR-21 binds TLR8 in endosomes/lysosomes, activates ERK, and promotes CCL2 expression, which increases Nav1.8 channel activity and facilitates neuronal excitability (21). Such a miR-21–dependent but indirect mechanism could explain the reduction in CCL2 expression that we detected in miR-21–cKO neurons.

Furthermore, lack of miR-21 in neurons is associated with upregulation of TGF-β pathway components in macrophages, which acquire an M2/tissue-repair-like phenotype. Since TGF-βR2 and TGF-β1 are known targets of miR-21, it is feasible that miR-21 transferred from injured neurons to macrophages maintains a proinflammatory phenotype via suppression of such components of this antiinflammatory pathway. Thus, these data indicate that neuronal miR-21 is a key player in the regulation of neuro-immune communication in DRGs and that miR-21 can be inhibited in macrophages to attenuate neuropathic pain.

Our search for neuron-derived miR-21 targets in macrophages has been guided by our genome microarray analyses that identified the TGF-β–related pathway as a potential target of neuronal miR-21. Consistent with this bioinformatics indication, in primary macrophages mimic-21 and antagomir-21 downregulate and upregulate *Tgfb1* and *Tgfbr2* expression, respectively, while antagomir-21 increases TGF-β1 secretion and favors polarization toward an antiinflammatory phenotype via upregulation of *Il10* and downregulation of *Tnfa* and *Il6*. Moreover, antagomir-21 activates the TGF-β canonical pathway via the SMAD family and not the ERK or p38 pathway, suggesting that miR-21 selectively inhibits TGF-β signaling in macrophages.

TGF-β is a pleiotropic and potent antiinflammatory cytokine, and we suggest that neuron-derived miR-21 exerts a pronociceptive action via inhibition of the macrophage TGF-β pathway. This is congruent with evidence that intrathecal administration of TGF-β1 inhibits development of neuropathic allodynia (24) to a similar extent as miR-21 silencing in sensory neurons, and intrathecal injection of bone marrow stromal cells, which release TGF-β1 (25), reduces neuropathic hypersensitivity to a similar extent to BMDMs transfected with antagomir-21. In DRGs, TGF-β1 likely acts via inhibition of proinflammatory cytokine expression (24, 33). In this context, our evidence shows that intrathecal delivery of antagomir-21–transfected BMDMs, which express high levels of TGF-βR2 and secrete TGF-β1, exerts prompt anti-allodynic action in neuropathic conditions. We think it is plausible that TGF-β1 would activate TGF receptors in macrophages to maintain an M2-like phenotype via suppression of proinflammatory cytokines. However, we do not exclude a possible effect of TGF-β1 in sensory neurons, which express both TGF-βR1 and TGF-βR2 (34), though the effects of this cytokine in peripheral neurons are both complex and model dependent (35). Nevertheless, TGF-β1 shows unequivocal antinociceptive effects in neuropathic pain models (24), evidence that is indirectly supported by our data that also highlight Spry2 protein regulation (36) rather than TGF-β modulation as a possible miR-21 effect on neuronal regeneration. Macrophages’ contribution to the development and maintenance of neuropathic pain is the result of these cells being highly plastic and critical at initiating inflammation and fostering tissue repair, depending on their origin and tissue environments (5). The commonly described M1- and M2-like states oversimplify macrophage heterogeneity and do not fully reflect the vast array of functions that can be adopted during disease and injury. Despite these limitations, the M1-/M2-like phenotype distinction offers valuable insights into investigating macrophage phenotype–dependent processes (37). For instance, intrathecal administration of M2-like macrophages inhibits osteoarthritis-like pain (38, 40), while M1-like macrophages exacerbate inflammatory pain (18). Conversely, macrophage depletion reduces neuropathic allodynia (12). Here, we report that antagomir-21–treated macrophages reverse neuropathic allodynia, express higher levels of TGF-βR2, and engraft and shift the pool of DRG macrophages toward a CD206^+^MHCII^–^ M2-like phenotype.

Intriguingly, antagomir-21–treated macrophages injected intrathecally accumulate in L3-L4-L5 DRGs and our most plausible explanation is that miR-21 inhibition at least partially promotes macrophage survival. Indeed, tumor suppressors are miR-21 targets, as miR-21 inhibits apoptosis and promotes survival of cancer cells (39, 40). However, inconsistencies have emerged since, for example, miR-21 deficiency in macrophages inhibits NLRP3 inflammasome expression, NF-κB activation, and IL-1 secretion, resulting in the reduction of pyroptosis (41). Conversely, overexpression of miR-21 increased cell invasion while decreasing apoptosis of fibroblast-like synoviocytes (42). Such discrepancies can be explained by (a) the type of cells, (b) conditions used for alteration of miR-21 expression, and (c) duration and context of the alteration.

Of interest, miR-21 silencing in sensory neurons is associated with lower levels of CCL2 and reduced accumulation of monocytes/macrophages expressing CCR2 in DRGs. Whether DRG macrophages derive from circulating monocytes or proliferation of resident macrophages is still a matter of debate (12, 43, 44). Nevertheless, in our experimental settings, we suggest that miR-21 silencing in neurons reduces circulating monocyte infiltration rather than resident macrophage proliferation since we observe a reduction in CCR2 expression and no alteration of Ki67^+^F4/80^+^ cell numbers.

Here we advanced our understanding of miR-21–specific mechanisms and targets, as we strengthened evidence that silencing miR-21 in sensory neurons attenuates development and maintenance of neuropathic allodynia and show that such an antinociceptive effect is mediated by TGF-β in DRG macrophages.

Our results support miR-21 inhibition as a strategy to reduce neuropathic pain through maintenance of DRG macrophage polarization in the M2-like state and highlight the important role of the neuro-immune communication that contributes to nociceptive mechanisms.

In conclusion, the findings of this preclinical research may have direct relevance to a substantial clinical problem and provide important evidence for the therapeutic potential of noncoding RNAs as new targets for the treatment of neuropathic pain, perhaps in combination with established therapeutics such as gabapentin, to obtain optimal analgesic efficacy (45). Clinical relevance of noncoding RNAs has been recently highlighted by the first-in-human miRNA-based phase I therapeutic trial in patients with liver cancer (ClinicalTrials.gov NCT01829971), which utilized miRNA mimetics encapsulated in a nanoparticle-based formulation that has shown minimal side effects to date. Our preclinical evidence on the effectiveness of modulating mir-21 levels to mitigate neuropathic nociception may lead to a clinical trial on the analgesic effect of nanoparticles containing antagomir-21 in patients with neuropathic pain.

## Methods

### Animals.

All studies were conducted in C57BL/6 black male and female mice (Charles River). Animals were housed under a 12-hour light/12-hour dark cycle with ad libitum access to food and water. miR-21 conditional mutant mice with a null first conditional allele were crossed with Advilin-Cre driver mice for conditional ablation in sensory neurons (16). Adult 8- to 12-week-old miR-21–cKO mice and their control littermates were randomly assigned to groups. Each group contained the same number of age-matched mice of both sexes.

### Induction of peripheral neuropathy.

Mice were subjected to the SNI model of neuropathic pain, as previously described (46). Briefly, under 2.5% isoflurane anesthesia, a small incision was made in the skin and muscle of the left thigh, and then the sciatic nerve and its 3 terminal branches were exposed. The common peroneal and tibial nerves were located, and the distal nerve stump was removed, leaving the sural nerve intact. Sham operations were performed by exposing the sciatic nerve without excision.

### TGF-βR1 inhibitor administration.

TGF-βR1 inhibitor (SB431542) was purchased from Selleckchem (S11067). Intrathecal injection was performed on both WT and miR-21–cKO mice on day 5 after SNI under light isoflurane anaesthesia, as previously described (47). Briefly, a spinal cord puncture was made using a 30-gauge needle and Hamilton syringe between L4 and L5 levels to deliver 100 pmol per mouse.

### Behavioral testing.

Mechanical thresholds were measured by applying calibrated von Frey monofilaments (0.008–1.0 g) to the plantar surface of the hind paw. Mice were placed in individual compartments, and all tests began after 30 minutes of habituation during the light cycle. The testing started with the application of a 0.07-g filament until the paw was withdrawn in a reflex unrelated to movement or grooming. Filaments were applied to the left and right hind paws alternately. Fifty percent withdrawal thresholds were obtained using the up and down method, which is based on identifying a positive or negative response with the 0.07-g filament; if a response is observed, a lower force is applied and vice versa until a change in response is observed or the application of the 1-g filament fails to induce a response (48). The 50% paw withdrawal thresholds were calculated using Dixon’s method (49).

### Primary macrophage culture.

BMDMs were isolated and generated as previously described (50). Briefly, bone marrow cells were obtained by flushing femurs and tibias of adult mice. A single-cell suspension was obtained by passing cells through a 70-μm cell strainer. Cells were then cultured in 10-cm non–tissue culture–treated dishes for 7 days in Dulbecco’s modified Eagle’s medium (DMEM; Gibco) supplemented with 10% (v/v) heat-inactivated fetal bovine serum (FBS HI; Gibco), 10% (v/v) L929 cell–conditioned medium as a source of macrophage colony stimulating factor (51), and 1% (v/v) penicillin/streptomycin (Gibco/Thermo Fisher Scientific) at 37°C and 5% CO_2_. The macrophage enrichment was validated by flow cytometry using F4/80 and CD11b as markers.

For PMs, mice were euthanized, and lavage of their peritoneal cavity was performed with 10 mL of ice-cold PBS (Sigma-Aldrich)/2 mM EDTA (Invitrogen). Peritoneal cells were centrifuged, resuspend in DMEM, 10% FBS, and 1% (v/v) penicillin/streptomycin and seeded in Petri dishes for a minimum of 2 hours to allow the adhesion of macrophages.

### Macrophage transfection.

Macrophages (1 × 10^6^) were transfected using FAM-labeled miR-21-5p mimic or control-N5 (1 μg; Qiagen) for mimic-21 experiments, and miR-21-5p antagomir or scramble control (1 μg; Qiagen) for antagomir-21 experiments. The transfection was performed using Lipofectamine 3000 reagent (Invitrogen, L3000-0115) following the reverse transfection method. Transfected cells were cultured for 48 hours at 37°C and 5% CO_2_. Culture media were then removed, and TGF-β levels (pg/mL) were quantified using ELISA kits (Abcam, ab119557), according to the manufacturer’s instructions. Cell lysates were obtained using a lysis/binding buffer provided by the mirVana miRNA Isolation Kit (Invitrogen, AM1561). Total and small RNAs were isolated, and miRNA levels were detected by quantitative polymerase chain reaction (qPCR). For flow cytometry analysis, cells were detached using cell scrapers (Starlab), centrifuged at 300*g* for 10 minutes, and then resuspended in FACS buffer (0.5% BSA and 2 mM EDTA in PBS).

### DRG neuron culture.

DRGs from miR-21–cKO mice and their littermate controls were collected and placed into Ham’s F-12 Nutrient Mixture (Gibco). DRGs were then dissociated using 3 mg/mL Dispase (Roche), 0.1% collagenase (Sigma-Aldrich), and 200 U/mL DNase I (Sigma-Aldrich) in F-12 medium (Gibco). Thereafter, DRGs were triturated, and cell suspensions centrifuged at 300*g* for 6 minutes. Pellets were resuspended in fresh DRG medium and plated on glass coverslips precoated with poly-L-ornithine (100 μg/mL; Sigma-Aldrich) and laminin (40 μg/mL; Roche). Cultures (10,000 cells/well) were incubated at 37°C for 24 hours, and then stimulated with vehicle or capsaicin (1 μM) for 3 hours. Culture media were removed, and CCL2 was measured using ELISA (R&D Systems, DY479-05) in DRG neuronal culture media, according to the manufacturer’s instructions.

Transmigration assay was performed using cell culture inserts (Transwell plate, Costar) with 8-μm porous polycarbonate filters. DRG neurons (10,000) were cultured in 300 μL F-12/10% FBS in the lower compartment for 24 hours, and then 40,000 WT BMDMs treated with vehicle or CCR2 antagonist (1 μM; Merck) were added to the upper filter. DRG neurons were then stimulated with capsaicin (1 μM) for 3 hours.

### Real-time PCR.

Total and small RNAs were isolated using a mirVana miRNA Isolation Kit (Invitrogen) according to the manufacturer’s instructions and RNA eluted using RNase-free water. Both concentration and purity were measured by a spectrophotometer (NanoDrop ND-100, Labtech). The total RNA samples (100 ng) were reverse transcribed with a Quantitec Reverse Transcription Kit (Qiagen). Real-time PCR was performed using SYBR Green I Master Mix (Roche) and specific primers for mouse genes (Qiagen) in a LightCycler 480 (Roche). Duplicate CTs were averaged, and results analyzed by the 2^−ΔΔCT^ method using *18S* or *Actb* as a housekeeper gene. For miRNA detection, 5 ng/μL was used from each small RNA template, and cDNA synthesized using the miRCURY LNA Universal cDNA Synthesis kit II (Qiagen). Real-time PCR for miR-21-5p, -155, and -706 was performed using SYBR Green I Master Mix in a LightCycler 480. Duplicates of CTs were averaged, and the relative quantities of miRNAs calculated using the 2^−ΔΔCT^ method and normalized to several artificial spike-ins as controls for extracellular miRNAs.

### Flow cytometry.

Mice were deeply anesthetized by intraperitoneal injection of pentobarbital (Pentoject) and perfused with ice-cold PBS to remove circulating blood from the vasculature. DRGs and sciatic nerves were rapidly harvested and placed into ice-cold HBSS (Gibco). Single-cell suspensions were obtained after enzymatic digestion using 3 mg/mL Dispase (Roche), 0.125% collagenase (Sigma-Aldrich), and 200 U/mL DNase I (Sigma-Aldrich) in F-12 medium (Gibco) for 30 minutes at 37°C, followed by centrifugation at 300*g* for 10 minutes and resuspension in FACS buffer. Samples were stained for viability with Live/Dead Fixable Near IR (Invitrogen, L10119) for 30 minutes, followed by staining with directly conjugated antibody mix.

The following antibodies were used: anti–mouse CD16/CD32 (clone 93; BioLegend, 101302), Brilliant Violet 605 (BV605)–conjugated anti-CD45 (clone 30-F11; BioLegend, 103139), BV421-conjugated anti-CD11b (clone M1/70; Biolegend, 101235), allophycocyanin (APC)-conjugated anti-F4/80 (clone BM8; BioLegend, 123116), PercP-Cy5.5–conjugated anti-MHCII (clone AF6-120.1; BioLegend, 116416), phycoerythrin (PE)-conjugated anti–TGF-βR2 (R&D Systems, FAB532P), PE-Cy7–conjugated anti-CD206 (clone C068C2; BioLegend, 141720), FITC-conjugated anti-CD206 (clone C068C2; BioLegend, 141704), FITC-conjugated anti-Ly6C (clone HK1.4; BioLegend, 128006), PE-conjugated Ly6G (clone 1A8; BioLegend, 127607), and PE-Cy7–conjugated anti-CCR2 (CD192; clone SA203G11; BioLegend, 150611). All antibodies were used at a dilution of 1:100, except for PE-conjugated anti–TGF-βR2, which was used at 1:50. Macrophages were identified as CD45^+^CD11b^+^F4/80^+^. M1-like macrophages were identified as CD45^+^CD11b^+^F4/80^+^MHCII^+^CD206^–^. M2-like macrophages were identified as CD45^+^CD11b^+^F4/80^+^MHCII^–^CD206^+^. Monocytes were identified as CD45^+^CD11b^+^Ly6C^+^Ly6G^–^. Classical monocytes were identified as CD45^+^CD11b^+^Ly6C^hi^Ly6G^–^. Nonclassical monocytes were identified as CD45^+^CD11b^+^Ly6C^lo^Ly6G^–^. Neutrophils were identified as CD45^+^CD11b^+^Ly6C^+^Ly6G^+^. The total number of cells was then normalized to counting bead number (BioLegend, 424902). Cells were analyzed using a flow cytometer (LSR II, BD Biosciences).

### Western blotting.

Macrophages (1 × 10^6^ cells) and L3-L4-L5 DRGs were lysed in RIPA buffer (Sigma-Aldrich) supplemented with antiphosphatase (Phostop, Roche) and protease inhibitor (Roche). Protein concentration was determined by BCA assay (Bio-Rad) prior to denaturation. Samples were loaded into 10% SDS-PAGE gels and transferred onto polyvinylidene difluoride (PVDF) membranes. Membranes were probed with the following primary antibodies: rabbit anti-SMAD4 (1:1000; Cell Signaling Technology, 46535), rabbit anti–p-ERK (1:1000; Cell Signaling Technology, 9101), rabbit anti–p-p38 (1:1000; Cell Signaling Technology, 9211), rabbit anti-ERK (1:1000; Cell Signaling Technology, 4695), rabbit anti-p38 (1:1000; Cell Signaling Technology, 9212), and rabbit anti-CCL2 (1:1000; Invitrogen, MAS-17040). GAPDH (1:2000; Abcam, ab8245) and β-actin (1:1000; Cell Signaling Technology, 4967) were used as loading controls. Results were visualized with horseradish peroxidase–coupled anti–rabbit immunoglobulin (Dako, Agilent) using enhanced chemiluminescence detection reagents. Protein abundances were analyzed by densitometry scanning using Fiji (ImageJ 1.52i, NIH)

### Immunofluorescence.

For tissue immunofluorescence, mice were transcardially perfused with ice-cold PBS, and lumbar spinal cords and L3-L4-L5 DRGs were harvested and placed into 4% paraformaldehyde (PFA) (Sigma-Aldrich). Transverse sections of the spinal cord (20 μm) and DRG (10 μm) were taken using a cryostat (Bright Instruments). Sections were postfixed for 10 minutes, permeabilized with PBS/0.1% Triton X-100, and then incubated with rat anti–mouse F4/80 (1:200; Abcam, ab6640), followed by anti-rabbit–Alexa Fluor 546 secondary antibody (1:1000; Invitrogen, A11081), Ki67 (1:250; Abcam, ab16667), CCL2 (1:500; Invitrogen, MAS-17040), and IB4 (1:500; Invitrogen, I32450). For macrophage and DRG culture immunofluorescence, cells were plated on Labtec chamber slides (Thermo Fisher Scientific), fixed with 4% PFA, and then permeabilized with PBS/0.1% Triton X-100 for 10 minutes. The following antibodies were used: rabbit anti–mouse p-SMAD2/3 (1:1000; Cell Signaling Technology, 8828), rat anti–mouse F4/80 (1:200; Abcam, ab6640), rabbit anti–mouse TGF-βR2 (1:100; R&D Systems, FAB532P), rabbit anti–β-III tubulin (1:1000; Thermo Fisher Scientific, MA1-118), followed by fluorophore-coupled secondary antibodies (1:1000, Alexa Fluor 568, 488, 647; Invitrogen). The immunoreactivity was captured using a Zeiss LSM710 confocal microscope and images were acquired using the LSM software (Zeiss).

### Macrophage isolation from the DRG.

L4/L5 DRGs from miR-21–cKO mice and their littermate controls were harvested and digested as previously described (16). The single-cell suspensions were magnetically labeled with anti-F4/80 MicroBeads Ultrapure (Miltenyi Biotec, 130-110-443) for 15 minutes at 4°C, and then loaded onto a MACS column (Miltenyi Biotec, 130-042-401) placed on the magnetic field separator. The F4/80^+^ cells (positive fraction) were retained within the column and the unlabeled cells depleted in the F4/80^+^ (negative fraction) run through. The F4/80^+^ cells were eluted from the columns in PBS, 0.5% BSA, and 2 mM EDTA, followed by a centrifugation at 300*g* for 10 minutes.

### Extracellular vesicle isolation and analysis.

Supernatants from cultured DRG neurons of miR-21–cKO mice and their littermate controls were collected and centrifuged at 13,000*g* for 2 minutes for depletion of apoptotic bodies and cell debris. Supernatants were further incubated with CellTrace far-red dye (1 μM, Thermo Fisher Scientific, C3456) for 10 minutes on ice. Samples were then ultracentrifuged at 100,000*g* for 1 hour at 4°C. Extracellular vesicles (EVs) were collected and analyzed using ImageStream, as previously described (16). Briefly, EV samples were run on the ImageStream under slow-speed flow and ×60 magnification, with the 658 nm laser set at 200 mW and the side scatter at 70 mW. Data are expressed as EV/mL.

### Intrathecal injection of macrophages.

BMDMs were intrathecally injected under light isoflurane anesthesia, as previously described (47).

For the M2-like experiment, BMDMs from *Cx3cr1^GFP/+^* mice were stimulated for 16 hours with IL-4 (R&D Systems) and TGF-β (R&D Systems). M2-like BMDMs were then lifted from the plates and resuspended in ice-cold PBS, followed by 3 washes prior to intrathecal injection of 5 × 10^5^ cells/5 μL per mouse. For the antagomir-21–treated macrophage experiment, BMDMs obtained from C57BL/6 mice were transfected with either antagomir-21 or scramble control for 48 hours, followed by 3 PBS washes prior to the intrathecal injection of 5 × 10^5^ cells/5 μL per mouse.

### Genome-wide microarray.

Macrophages (F4/80^+^CD11b^+^, 2–5,000 cells) were sorted from a pool of ipsilateral L4/L5 DRGs of SNI WT and miR-21–cKO mice using a FACSAria II sorter (BD Biosciences) or cultured PMs. Total RNA was prepared from the cell lysates. Each condition was represented by independently collected biological triplicates. Labeled cell extracts were processed for microarray analysis using the WT Pico Amplification kit (Thermo Fisher Scientific) and hybridized to Affymetrix Mouse 430V2 Arrays. The quality of cDNA and fragmented cDNA was assessed in an Agilent Bioanalyzer 2100. All analyses were performed in R (v4.2.0). Statistically significant differences between groups were determined using the affy R package (52). The parameters were set to RMA background correction and quantile normalization, with pm correct pmonly and amedianpolish. Significant differential expression was inferred based on a *P* value of less than 0.05. Enrichment for GO terms for individual comparisons was performed using the EnrichGO function from the clusterProfiler R package in Bioconductor. A *P*-value and *q*-value cutoff of 0.05 was used.

### Data availability.

All data generated in this study are included in the manuscript. In vivo and in vitro microarray data are deposited into the NCBI Gene Expression Omnibus (GEO) database under accession numbers GSE104270 and GSE227608, respectively.

### Statistics.

All data are expressed as mean ± SEM. Sample size is stated in the figure legends and was determined according to previous internal data/publications. Data analyses were performed using GraphPad Prism (v.9.0.1) by unpaired Student’s *t* test (2 groups), 1-way ANOVA followed by Tukey’s multiple-comparison test (3 or more groups), or 2-way ANOVA followed by Tukey’s multiple-comparison test (behavioral data). *P* values of less than 0.05 were considered significant.

### Study approval.

All mouse studies were conducted under ethical approval from the local animal guide for care and use of laboratory animals (Biological Services Unit at King’s College London), and in accordance with the Home Office regulations (Guidance on the Operation of Animals, Scientific Procedures Act, 1986).

## Author contributions

LZ and MM conceptualized the study. LZ, RS, GSL, SAM, FP, SF, and DC developed methodology/experiments. LZ, RS, GSL, and MM carried out the investigation. LZ and MM wrote the original draft of the manuscript. All authors read and approved the final manuscript.

## Supplementary Material

Supplemental data

## Figures and Tables

**Table 1:**
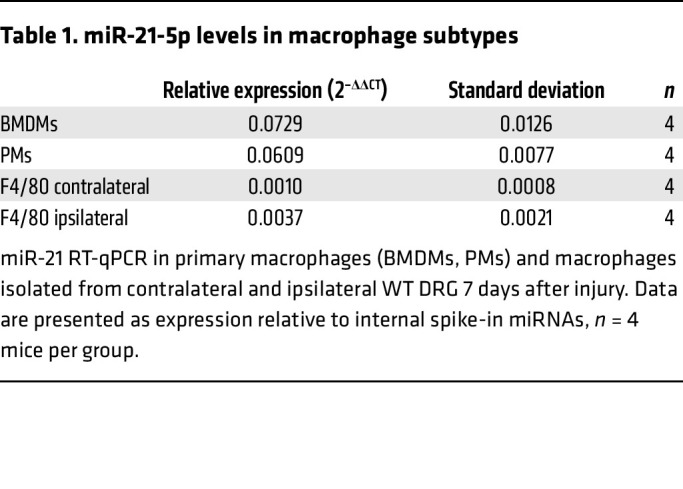
miR-21-5p levels in macrophage subtypes

## Version 1 04/18/2023

In-Press Preview

## Version 2 06/01/2023

Electronic publication
