# Supplementary material for: Silencing miR-21-5p in sensory neurons reverses neuropathic allodynia via activation of TGF-β–related pathway in macrophages
Source: J Clin Invest. 2023 Jun 1;133(11):e164472. doi: 10.1172/JCI164472 (PMC10232007; doi:10.1172/JCI164472)
Supplement: Supplemental data [file jci-133-164472-s232.pdf]

Silencing miR-21-5p in sensory neurons reverses neuropathic allodynia via activation of TGFB-related pathway in macrophages

Lynda Zeboudj, George Sideris-Lampretsas, Rita Silva, Sabeha Al-Mudaris, Francesca Picco, Sarah Fox, David Chambers, Marzia Malcangio.

Wolfson Centre for Age Related Diseases, King's College London, London SE1 1UL, UK.

Correspondence to Marzia Malcangio (marzia.malcangio@kcl.ac.uk) Wolfson Centre for Age Related Diseases, King's College London, London SE1 1UL, UK.

The authors have declared that no conflict of interest exists.

Supplementary Figures

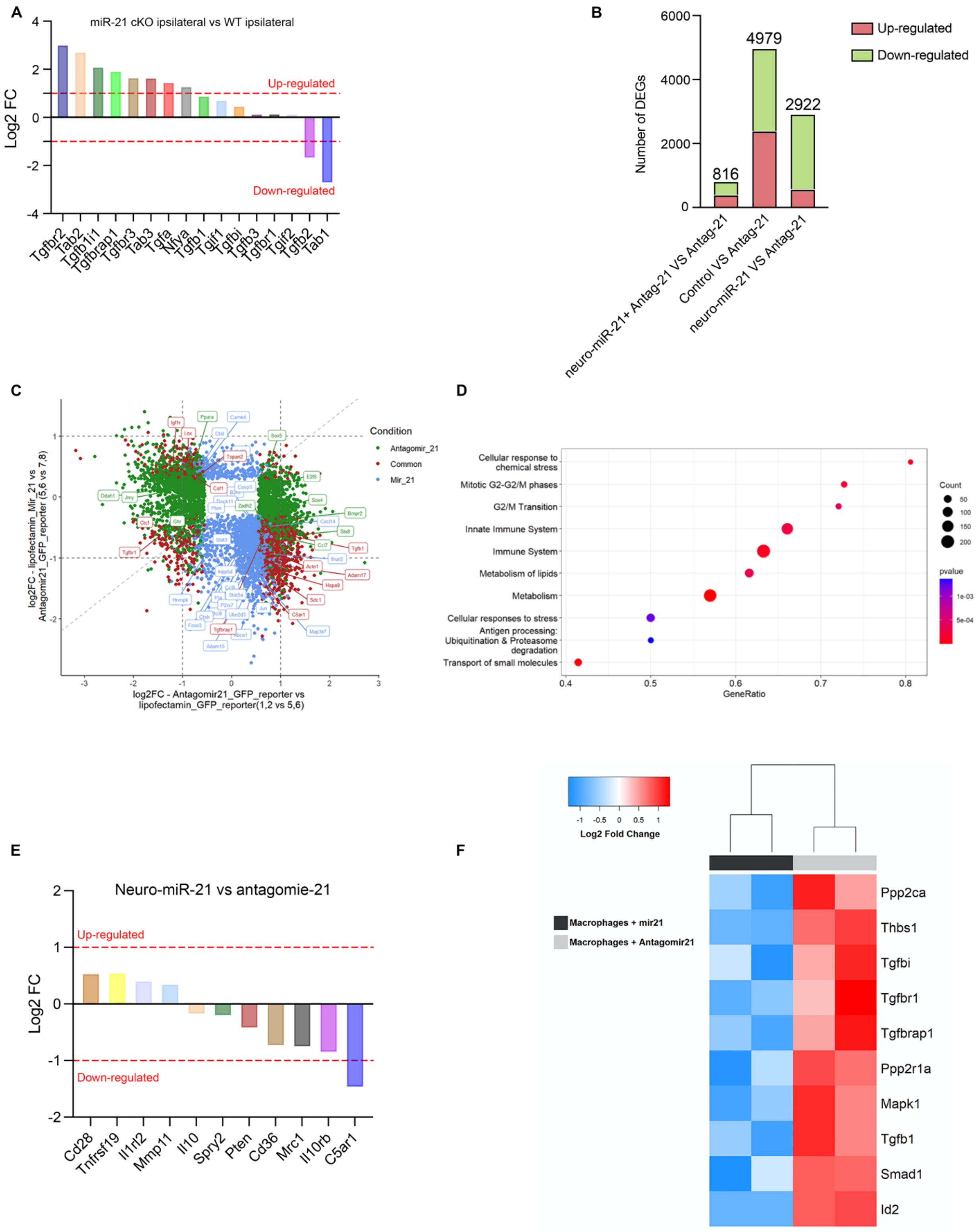

Supplementary Figure 1: **miR-21 induces a specific transcriptional profile in macrophages *in-vivo* and *in-vitro*.** Genome-wide microarray was performed on isolated macrophages ($F4/80^{+}CD11b^{+}$) from L4/L5 DRG of miR-21 cKO and WT control at 7 days after SNI **(A)** Bar charts represent the fold change of the *Tgfb*-related pathway gene changes in macrophages isolated from ipsilateral DRG of WT compared to miR-21-cKO ipsilateral DRG (n=5 mice/group). **(B)** Bar graphs represent the number of significantly up-regulated and down-regulated differentially expressed genes (DEGs) in peritoneal macrophages (PM) transfected with antagomir-21 exposed or not to neuronal exosomes overexpressing miR-21 (n=2, pooled from 6 mice in each replicate). **(C)** DEG comparison in PM transfected with miR-21 antagomir vs lipofectamine compared to PM transfected with miR-21 antagomir and exposed to neuron-derived miR-21. **(D)** Functional analysis of PM treated with antagomir-21 and exposed to neuron-derived miR-21. **(E)** Representative fold change of pro-inflammatory and anti-inflammatory genes in PM exposed to neuronal exosomes overexpressing miR-21 compared to antagomir-21 treated PM. **(F)** Heatmap showing the change of genes implicated in TGFB pathway in PM exposed to exosomes overexpressing miR-21 compared to antagomir-21 treated (n=2, pooled from 6 mice in each replicate).

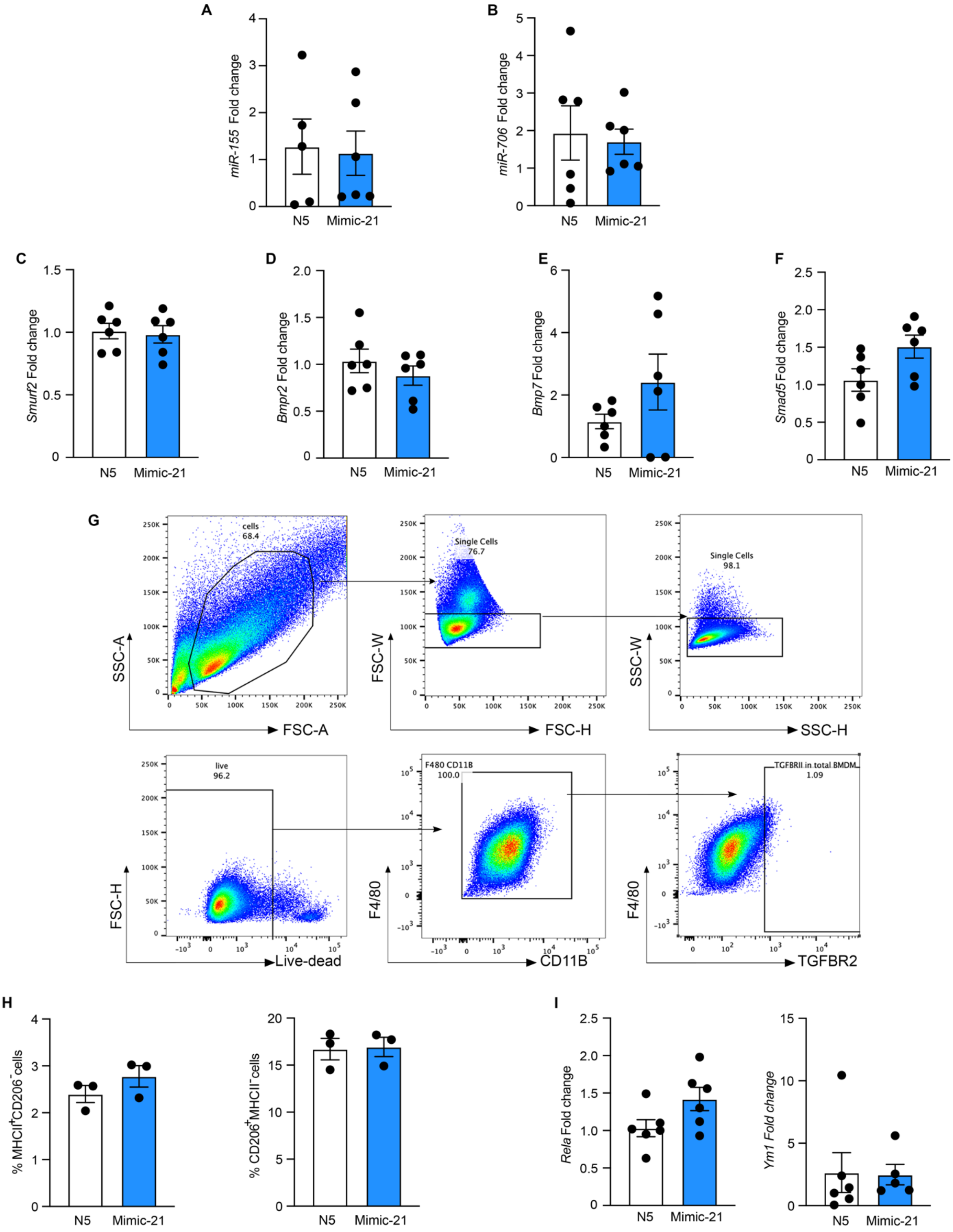

A
miR-155 Fold change
N5
Mimic-21
B
miR-706 Fold change
N5
Mimic-21
C
Smurf2 Fold change
N5
Mimic-21
D
Bmpr2 Fold change
N5
Mimic-21
E
Bmp7 Fold change
N5
Mimic-21
F
Smad5 Fold change
N5
Mimic-21
G
cells
68.4
SSC-A
FSC-A
Single Cells
76.7
FSC-W
FSC-H
Single Cells
98.1
SSC-W
SSC-H
live
96.2
FSC-H
Live-dead
F480 CD11B
100.0
F4/80
CD11B
TGFBRII in total BMDM
1.09
F4/80
TGFBR2
H
% MHCII+CD206- cells
N5
Mimic-21
% CD206+MHCII- cells
N5
Mimic-21
I
Rela Fold change
N5
Mimic-21
Ym1 Fold change
N5
Mimic-21

Supplementary Figure 2: miR-21 fosters a pro-inflammatory phenotype in macrophages and downregulates TGFB signaling.

(A) RT-qPCR of miR-155 and **(B)** miR-706 in PM after 48h of transfection with mimic-21 or N5 control (n=6). **(C)** RT-qPCR of *Smurf2*, **(D)** *Bmpr2*, **(E)** *Bmp7*, and **(F)** *Smad5* in PM at 48h after transfection with mimic-21 or N5 control (n=6), unpaired Student's *t*-test. **(G)** Flow cytometry gating strategy for TGFBR2 in the BMDM transfected with mimic-21 or N5 control. **(H)** Bar graphs show the percentage of $MHCII^{+}CD206^{-}$ and $CD206^{+}MHCII^{-}$ cells analyzed by flow cytometry, n=3 **(I)** RT-qPCR of *Rela*, and **(J)** *Ym1*, in PM transfected with mimic-21, n=5-6 per group. Data are presented as mean ± S.E.M.

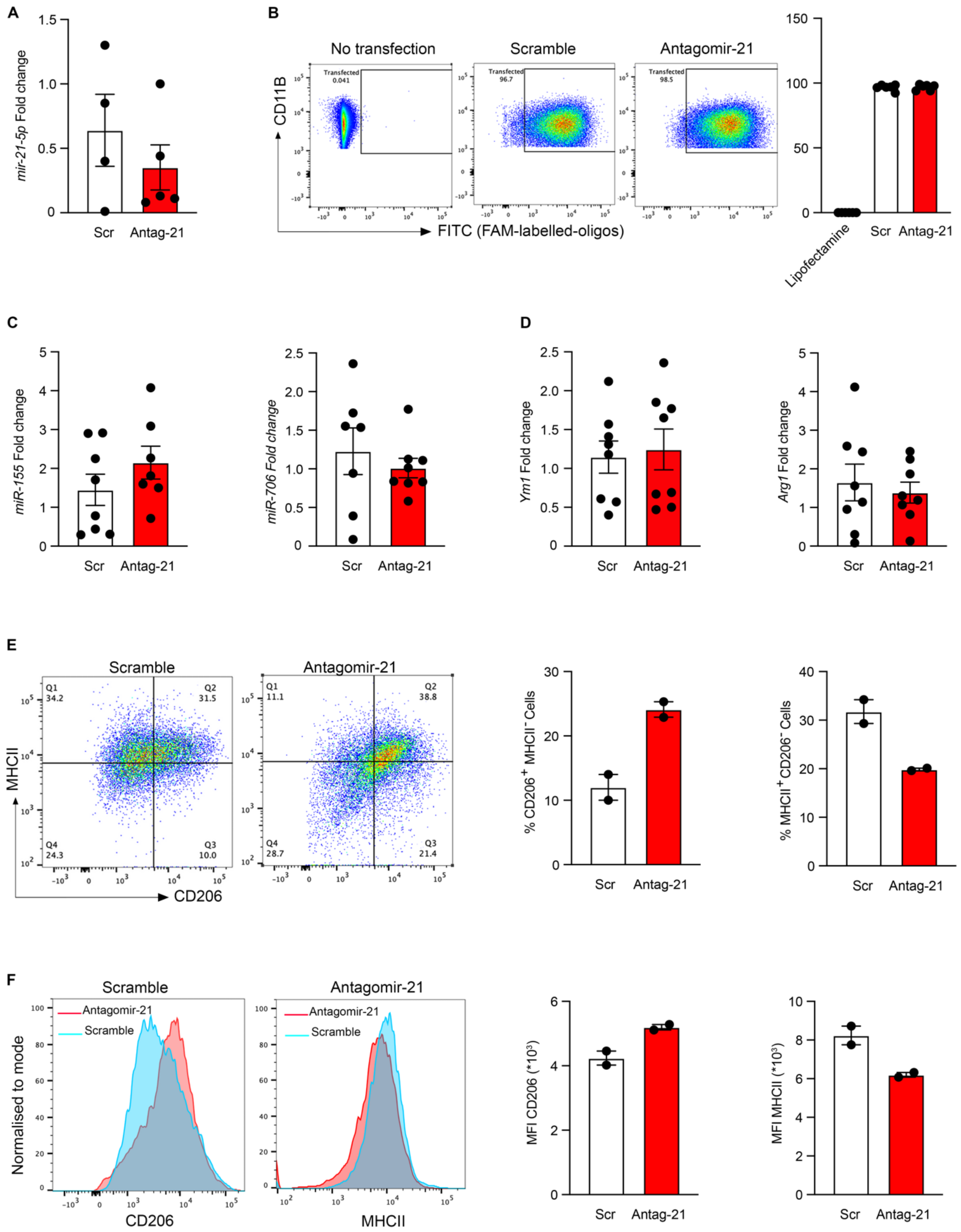

Supplementary Figure 3: miR-21 silencing in sensory neurons induces an anti-inflammatory phenotype of macrophages.

(A) miR-21-5p fold change in PM transfected with antagomir-21 or scramble control (n=4-5). **(B)** Flow cytometry analysis of antagomir-21 transfection efficiency in BMDM (n=4). **(C)** RT-qPCR of miR-155, miR-706 and **(D)** *Ym1*, *Arg1* in BMDM after miR-21-5p silencing (n=8). **(E)** Flow cytometry analysis of CD206, MHCII expression in BMDM transfected with antagomir-21 compared to scramble control (n=2 presented as mean of 2 independent experiments). **(F)** Representative histograms of CD206 and MHCII expression in BMDM transfected with antagomir-21 or scramble control by quantitative flow cytometry, the bar graphs represent MFIs of CD206 and MHCII respectively, (n=2 presented as mean of 2 independent experiments). Data are presented as the mean ± S.E.M.

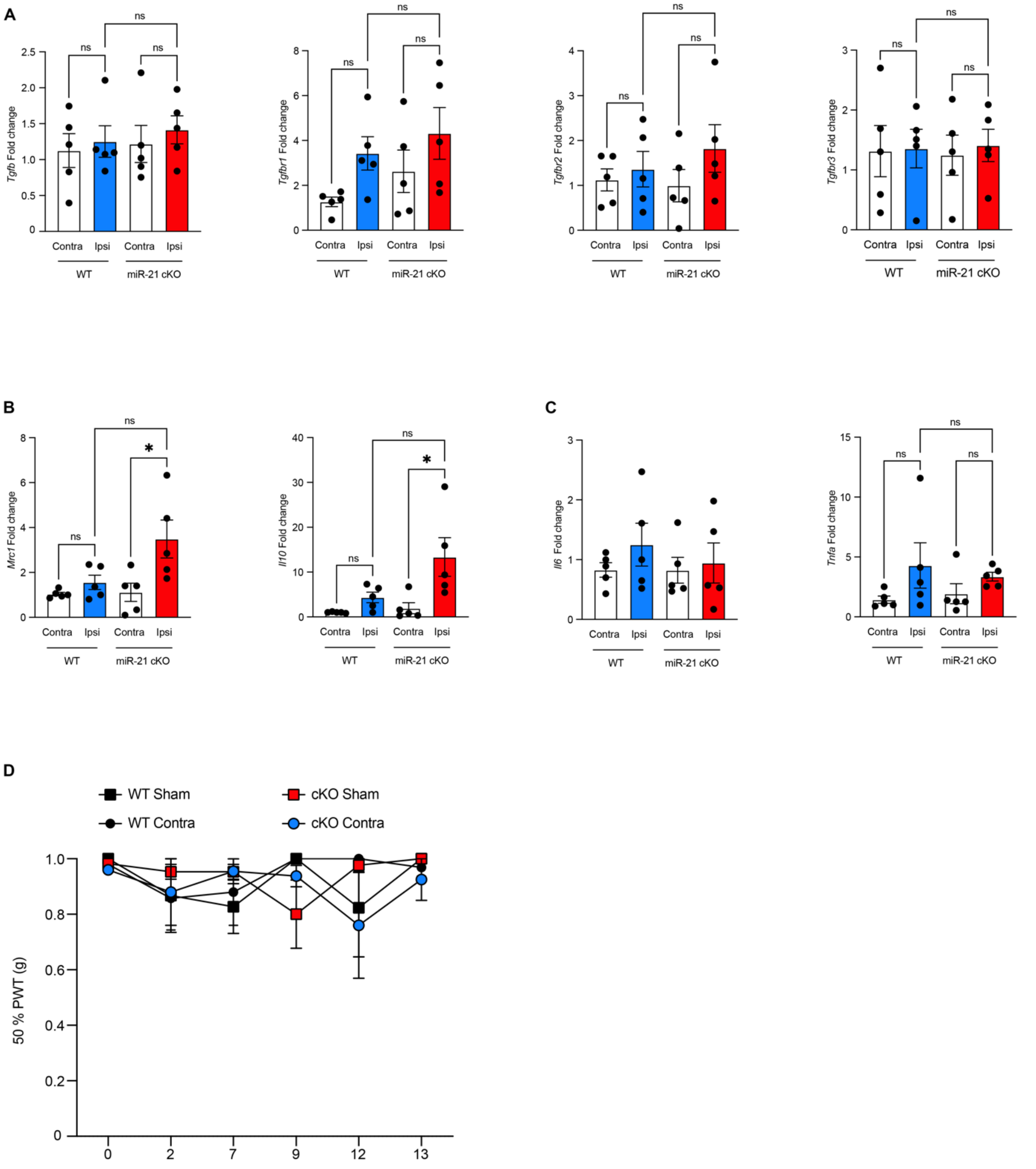

Supplementary Figure 4: miR-21 silencing in sensory neurons has no effect on TGFB-related pathway in DRG neurons. (A) RT-qPCR of *Tgfb*, *Tgfbr1*, *Tgfbr2*, *Tgfbr3* and in the negative fraction enriched in DRG neurons (F4/80$^-$), (n=5) **(B)** RT-qPCR of *Mrc1, Il10 and* ***(C)*** *IL6, Tnfa* in the positive fraction enriched in macrophages (F4/80$^+$), n=5, one-way ANOVA followed by Tukey's multiple comparison test. **(D)** miR-21 silencing in sensory neurons does not alter

contralateral thresholds in SNI compared to sham injuries (n=6). Data are presented as 50% PWT, Two-way ANOVA followed by Tukey multiple comparison test. Data are presented as mean ± S.E.M.

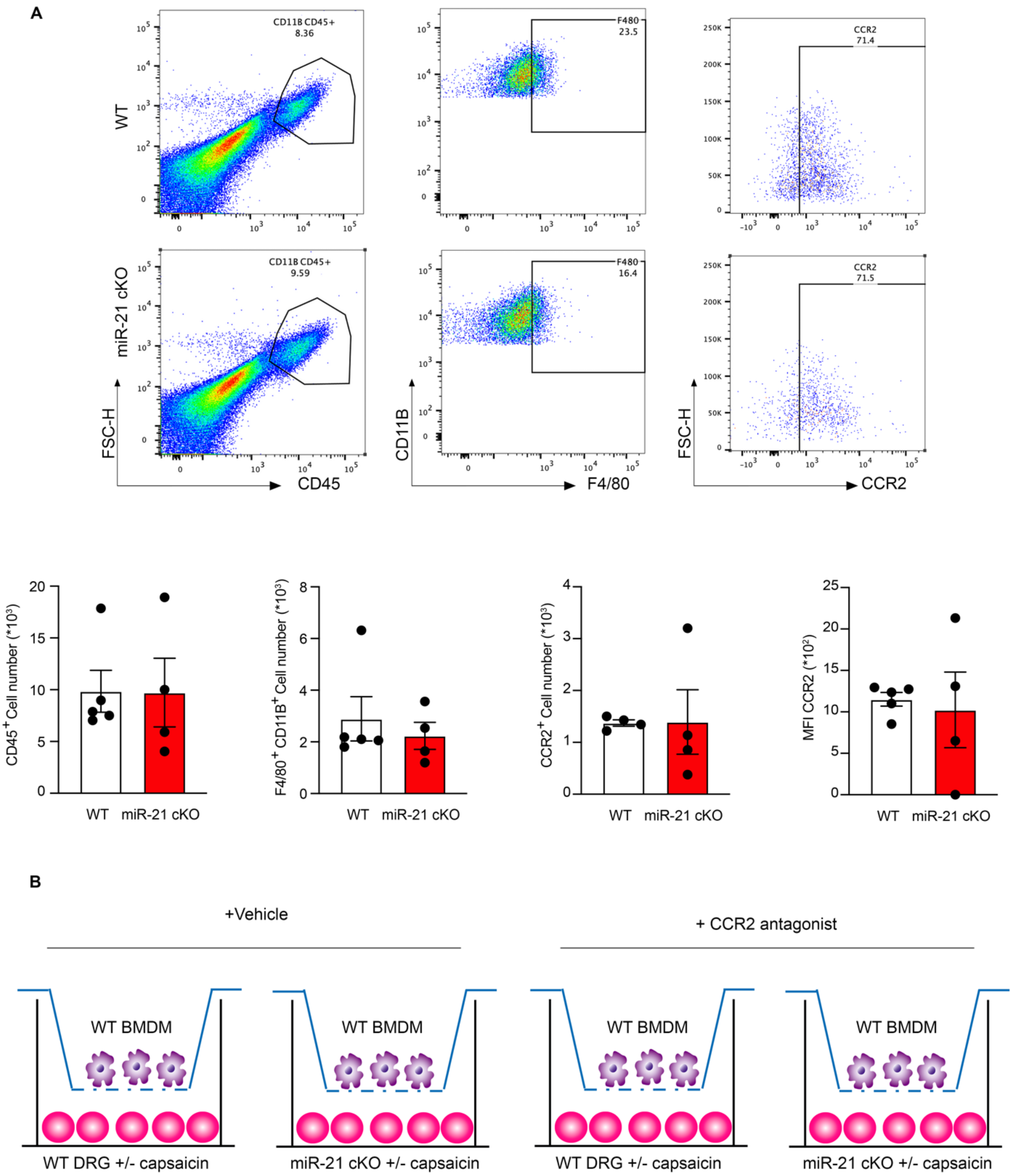

Supplementary Figure 5: miR-21 silencing in sensory neurons does not affect sciatic nerve macrophages.

(A) Flow cytometry analysis of $CD45^+$, $CD11b^+F4/80^+$, and CCR2 expression in WT and miR-21 cKO sciatic nerves at day 7 SNI (n=4), Data are presented as mean ± S.E.M. **(B)** Representative schematic of the Transwell™ design of DRG and macrophages trans-migration assay; WT and miR-21 cKO DRG neurons were cultured in the lower compartment stimulated with vehicle or capsaicin (1µM), and WT BMDM treated with vehicle or CCR2 antagonist were added to the top inserts.

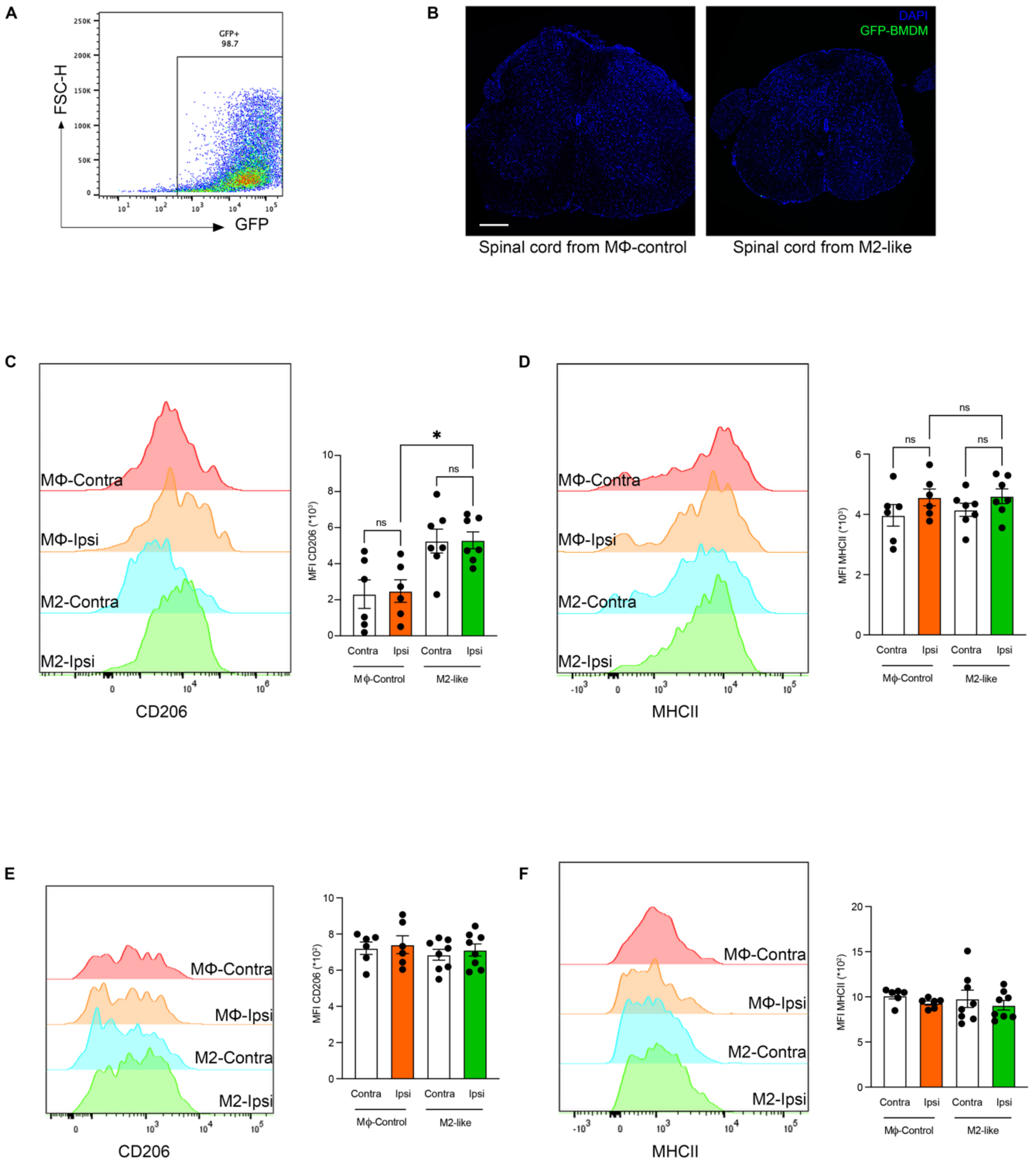

Supplementary Figure 6: Intrathecal delivery of M2-like macrophages induces CD206 expression in the sNAMs.

(A) Representative scatterplots of the injected GFP tagged BMDM. **(B)** Representative confocal images of cryo-sections of lumbar spinal cords at 2 h after i.t. injection of GFP$^+$ Mφ-control or M2-like macrophages, scale bar 100µm. GFP signal was not detected. **(C)** Representative

histograms of CD206 expression in DRG F4/80$^+$CD11b$^+$ at 2h after i.t injection, n=6-7, ns: not-significant, *p<0.05 one-way ANOVA followed by Tukey multiple comparison test. **(D)** Representative histograms of MHCII expression in DRG F4/80$^+$CD11b$^+$ at 2h after i.t injection n=6-7. **(E)** Representative histograms of CD206 expression in DRG F4/80$^+$CD11b$^+$ at 48h after i.t injection. **(F)** Representative histograms of MHCII expression in DRG F4/80$^+$CD11b$^+$ at 48h after i.t injection, n=6-8. Data are presented as mean ± S.E.M, one-way ANOVA followed by Tukey multiple comparison test.

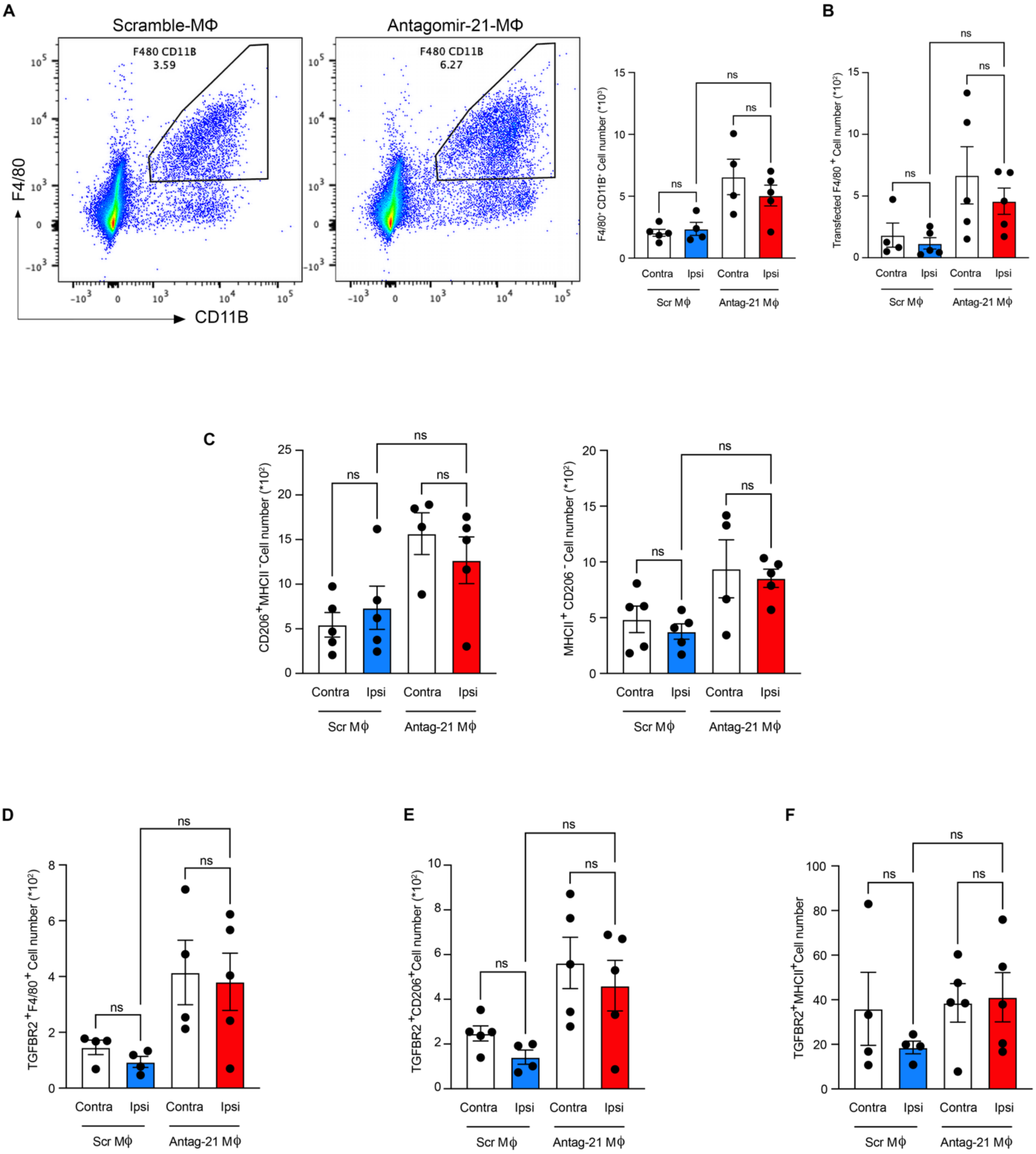

Supplementary Figure 7: Antagomir-21-BMDM tend to conserve M2-like phenotype at 48h after i.t. injection. (A) Representative scatterplots of $CD11b^+F4/80^+$ macrophages (gated on live cells) of ipsilateral DRG at 48h after i.t. injection of scrambled-BMDM or antagomir-21-BMDM, the bar chart represents the $F4/80^+CD11B^+$ absolute cell number, n=4-5, one-way

ANOVA followed by Tukey multiple comparison test. **(B)** Bar graph represents the number of the i.t. injected BMDM (FAM^+) at 48h after injection (n=5), one-way ANOVA followed by Tukey multiple comparison test. **(C)** Bar graph shows the absolute cell number of $CD206^+MHCII^-$, $MHCII^+CD206^-$ population in DRG at 48h after i.t. injection of scrambled-BMDM or antagomir-21-BMDM (n=4-5), one-way ANOVA followed by Tukey multiple comparison test. **(D)** Bar graph shows TGFBR2 expression in $CD11B^+F4/80^+$ in DRG at 48h after i.t. injection, n=4-5, one-way ANOVA followed by Tukey multiple comparison test. **(E)** Bar graph shows $TGFBR2^+CD206^+$ absolute cell number and **(F)** $TGFBR2^+MHCII^+$ population in DRG at 48h after the i.t injection. (n=4-5), one-way ANOVA followed by Tukey multiple comparison test. data are presented as mean ± S.E.M.
